# Unprecedented 2024 East Antarctic winter heatwave driven by polar vortex weakening and amplified by anthropogenic warming

**DOI:** 10.1038/s41612-026-01392-x

**Published:** 2026-04-01

**Authors:** Haosu Tang, Sihan Li, Julie M. Jones, Sergi González-Herrero, Andrew Orr, Friederike E. L. Otto, James A. Screen, Kyle R. Clem, Deniz Bozkurt, Jennifer L. Catto, Charlie C. Suitters, Michelle L. Maclennan, Yiming Sun

**Affiliations:** 1 School of Geography and Planning, University of Sheffield, Sheffield, UK; 2 WSL Institute for the Snow and Avalanche Research (SLF), Davos, Switzerland; 3 British Antarctic Survey, Cambridge, UK; 4 Grantham Institute, Imperial College, London, UK; 5 Department of Mathematics and Statistics, University of Exeter, Exeter, UK; 6 School of Geography, Environment and Earth Sciences, Victoria University of Wellington, Wellington, New Zealand; 7 Departamento de Meteorología, Universidad de Valparaíso, Valparaíso, Chile; 8 School of Electrical and Electronic Engineering, University of Sheffield, Sheffield, UK

**Keywords:** Climate sciences, Environmental sciences

## Abstract

During July–August 2024, East Antarctica experienced the most intense winter heatwave in the 46-year satellite era, with regional mean surface air temperatures across Dronning Maud Land exceeding the climatological mean by more than 9°C for 17 consecutive days. To explore the physical drivers and quantify the anthropogenic contribution to this unprecedented event, we propose a multi-model, multi-method attribution framework integrating regional climate model-based storyline attribution, circulation analogues, and large-ensemble probabilistic attribution. The results show that a pronounced weakening of the stratospheric polar vortex initiated a quasi-barotropic high-pressure anomaly, which enhanced meridional heat and moisture transport and accounted for approximately 50% of the observed surface warming. Across different models and attribution methods, synthesis of the attribution results indicates that anthropogenic warming intensified the event by approximately 0.7°C and more than doubled the likelihood of such exceptional winter heatwaves in the current climate. Probabilistic attribution further indicates that, compared to a natural climate without human influence, the likelihood of such events increases from 2–3 times today to ~6 times under moderate emissions and up to 26 times under high emissions by 2100. These findings reveal how human-induced warming is transforming even the coldest regions, with implications for ice shelf stability and predictability of future Antarctic extremes.

## Introduction

Antarctica plays a critical role in the global climate system and sea level regulation, with its vast ice sheet storing nearly 60% of the world’s total fresh water^1^. Among these, the East Antarctic Ice Sheet (EAIS) holds the largest reservoir of glacial ice, containing the equivalent of approximately 52 m of global sea level rise if completely melted^2,3^. Historically, the EAIS has been considered more stable than the West Antarctic and Greenland Ice Sheets because its bedrock largely lies above sea level and regional surface temperatures remain well below the melting point of ice. However, this assumed stability has been challenged by record-breaking heat extremes in recent years, implying the region’s susceptibility to rapid climate change. Although the EAIS has generally gained mass over the early 21st century (2000–2020)^4^, likely driven by more frequent extreme snowfall events, emerging evidence indicates that certain sectors of EA have begun to exhibit localized ice mass loss in recent decades (since approximately the 2000s–2010s) in response to atmospheric and oceanic warming^5–8^. The March 2022 EA heatwave was among the most striking examples of atmospheric warming over this region, with surface temperatures surging up to 40 °C above climatological norms, representing the largest temperature anomaly ever recorded in Antarctica^9,10^.

More recently, during the austral winter of 2024 (hereafter, seasons refer to those in the Southern Hemisphere), EA experienced another notable warming episode, with localized temperature anomalies in Dronning Maud Land (DML) substantially exceeding the seasonal average from July to August. Notably, several stations in DML recorded temperatures even approaching the melting point, which is exceptionally rare for mid-winter. This unseasonal warmth coincided with the earliest sudden stratospheric warming (SSW) on record in the Southern Hemisphere since the start of the satellite era in 1979, occurring in early July^11,12^. This warming had likely contributed to the persistently low sea ice extent off DML as well^13,14^, resulting in the second ever lowest annual maximum Antarctic sea ice extent in the satellite era, following the record low observed in 2023^15^. The atmospheric background was further shaped by a relatively weak Antarctic ozone hole, which developed later than average and ranked among the smallest observed in recent decades^13^. Together, these recent extreme events motivate the need to assess EA’s vulnerability to climate change and to disentangle the contributions of its large inherent climate variability, which is essential for improving projections of future changes in the Antarctic climate system.

Antarctic heatwaves are influenced by factors operating across multiple spatial scales. At the local scale, phenomena such as Foehn winds on the leeside of topographic barriers contribute significantly to extreme temperature fluctuations^16–19^. At the regional scale, weather systems, including blocking highs, cyclones, and atmospheric rivers, play a pivotal role in driving heatwave events^20–29^. For instance, the March 2022 EA heatwave was tied to an atmospheric river intrusion, channeled by a blocking high, which transported substantial amounts of heat and moisture deep into the continent^10^. These regional weather patterns are further modulated by remote climate drivers such as the stratospheric polar vortex and tropical–polar teleconnections, including the El Niño–Southern Oscillation (ENSO), primarily through their influence on the Amundsen Sea Low and the Southern Annular Mode (SAM)^30–35^. Additionally, anthropogenic climate change is increasingly affecting these remote drivers, adding further complexity to the behavior and evolving characteristics of Antarctic heatwaves^36–38^.

Human-induced climate change has unfolded a new pattern of more intense and frequent heat extremes worldwide in recent decades^39^, yet attribution of such extremes occurring in Antarctica remains limited^40–42^. Human-induced thermodynamic warming in Antarctica is often modulated by circulation‑driven dynamic variability that can either amplify or dampen local temperature responses^43^. This duality highlights the importance of employing complementary attribution methods to disentangle the contributions of thermodynamic versus dynamic contributors. Three principal approaches have been established in the extreme event attribution literature. Firstly, the probabilistic event attribution (PEA) method often applies statistical techniques to compare climate model simulations with and without anthropogenic forcing, thereby estimating changes in the probability or magnitude of extreme events^44^. Secondly, the circulation analogue approach quantifies the thermodynamic contribution by comparing present-day extreme event metrics with those from historical periods exhibiting similar large-scale circulation patterns^20^. When applied to the February 2020 Antarctic Peninsula heatwave, which featured regional mean temperature anomalies of 4.5 °C, this method attributes at least 0.4 °C of the event’s intensity to anthropogenic warming^16^. Thirdly, the storyline attribution treats circulation as conditionally fixed and focuses on how anthropogenic warming modifies the thermodynamic response under a given dynamical setup^45–48^. Recent application of the storyline-based attribution indicates that anthropogenic greenhouse gas (GHG) emissions amplified the March 2022 EA heatwave by approximately 2 °C^9^. These complementary methods are particularly valuable in Antarctica, where sparse observations, short instrumental records, pronounced internal climate variability, and model deficiencies in simulating sea ice complicate the assessment of extreme events^33,36^.

Here, we present a multi-model, multi-method attribution framework that integrates storyline, circulation analogue, and probabilistic approaches, combining process fidelity, flow sampling, and probability estimation. Applying this framework to the record-breaking July–August 2024 DML winter heatwave, we find that while rare blocking highs triggered by polar vortex weakening were the primary contributors, anthropogenic warming played a nontrivial role. Our results demonstrate that human-induced climate change has transformed what would have been a multi-century event under natural forcing into a centennial-scale occurrence in the present-day climate, providing a comprehensive assessment of both the underlying physical mechanisms and the anthropogenic contribution to this extreme Antarctic heatwave.

## Results

### Spatiotemporal characteristics of the 2024 winter heatwave

**Fig. 1:**
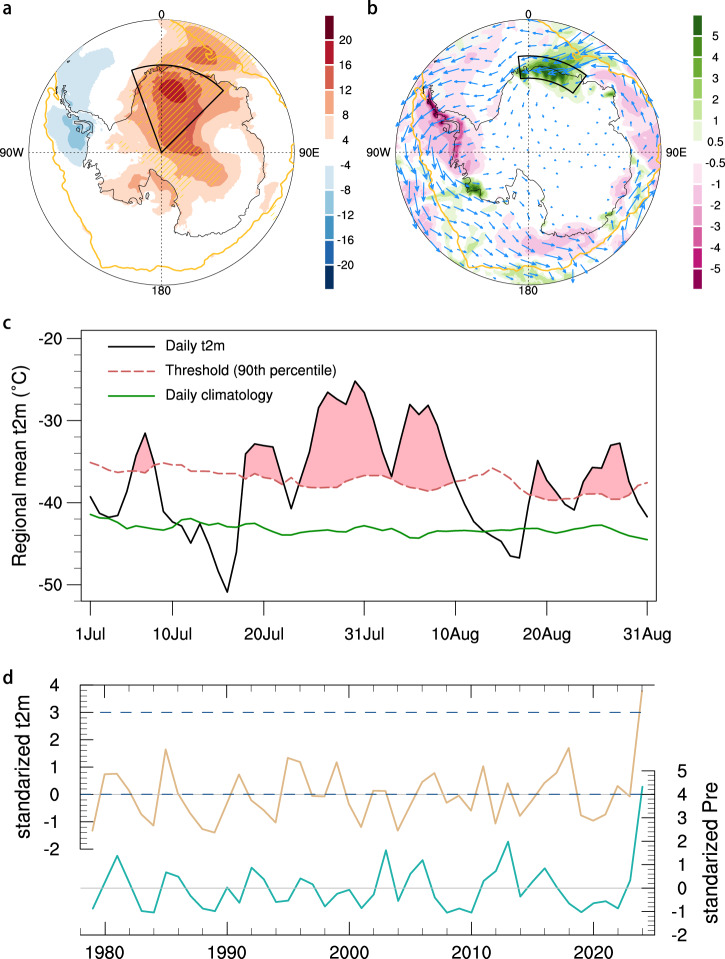
Spatial-temporary characteristics of 2024 East Antarctic winter heatwave. **a** T2m anomalies (shadings; unit: °C) during July 24 to August 9, 2024, relative to the 1981–2010 climatology. The black box indicates the Dronning Maud Land (20°W–45°E, 90–70°S). The grid points with the highest temperature since 1979 are hatched. The yellow contour indicates the Antarctic sea ice edge during the study period, defined as the boundary where SIC exceeds 15%. **b** Precipitation anomalies (shadings; unit: mm day^–^^1^) and vertically integrated moisture transport anomalies (vectors; unit: kg m s^–^^1^) during July 24 to August 9, 2024. The black box indicates coastal region with the most intense precipitation. **c** Daily T2m evolution averaged over the black box in (**a**) during mid-winter 2024. Black line denotes daily T2m, while dashed red line for 5-day running mean daily 90th threshold, and green line for daily climatology. **d** Time series of standardized, area-weighted regional mean T2m anomalies (pale gold) and precipitation anomalies (green) over the black boxes in (**a**) and (**b**), respectively, during 24 July–9 August for 1979–2024. The dashed lines show the 3- and 4-standard-deviation thresholds for two time series, respectively.

Figure 1a shows that the 2024 winter heatwave affected an extensive region across EA, with the most intense temperature anomalies centered over DML and extending offshore into the eastern Weddell Sea. The strongest near-surface 2-m air temperature (T2m) anomalies reached up to 27.5 °C in central DML on 5 August, as indicated by the European Centre for Medium-Range Weather Forecasts Reanalysis version 5 (ERA5). At the Relay and Dome Fuji stations, T2m anomalies peaked at 30.1 °C and 30.9 °C, respectively, both on 5 August (Supplementary Fig. 1). This intense warm anomaly was accompanied by anomalously heavy precipitation, particularly along the DML coast (Fig. 1b). The precipitation anomaly was primarily driven by enhanced moisture transport from the mid-latitudes, likely facilitated by a series of atmospheric rivers delivering increased water vapor to the region^49^, and further intensified by orographic uplift along the coastal terrain^50^. In contrast, the Antarctic Peninsula experienced anomalous cooling and drying, indicating a pronounced meridional dipole pattern in Antarctic surface meteorological anomalies (Fig. 1a, b), which was concomitantly reflected in sea ice changes, with reductions off DML and enhancements off the Antarctic Peninsula (Supplementary Fig. 2). Based on the heatwave definition of at least three consecutive days with daily T2m exceeding the 90th percentile of the local 1981–2010 climatology (see Methods), the 2024 DML heatwave occurred from 24 July to 9 August (Fig. 1c), with the regional mean T2m anomaly reaching 9.4°C (based on ERA5). Heatwave results derived from the Japanese Reanalysis for Three Quarters of a Century (JRA-3Q) dataset and individual DML weather stations (Supplementary Table 1) were consistent with ERA5-based findings, with both showing two pronounced peaks in late July and early August (Supplementary Fig. 1).

The regional mean T2m and precipitation during mid-winter 2024 reached the most extreme levels in the ERA5 record since the start of the satellite era in 1979, exceeding 3- and 4-fold standard deviations, respectively (Fig. 1d). Over the period 1979–2024, local T2m anomalies exhibit a statistically significant warming trend of 0.5 °C per decade (*p* < 0.05), superimposed on substantial interannual variability. To assess the rarity of the 2024 DML heatwave, we apply the non-stationary generalized extreme value (GEV) distribution to the regional-mean Tx17d index, which represents the maximum 17-day running mean T2m during mid-winter (July and August; see Methods). The return period of this heatwave is estimated at approximately 1-in-135 years in both ERA5 and JRA-3Q reanalysis, indicating its exceptional nature within the historical record (Supplementary Fig. 3).

**Fig. 2:**
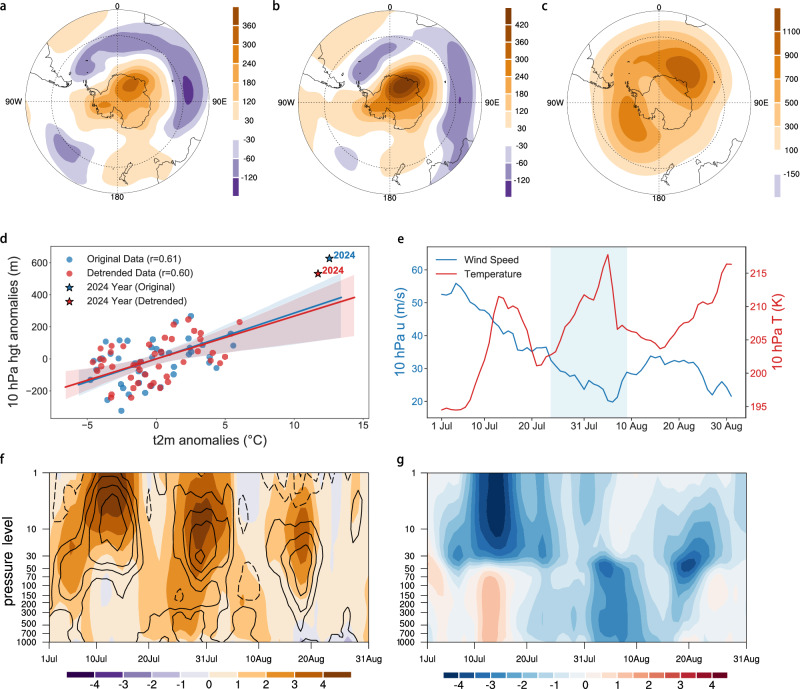
Atmospheric circulation anomalies associated with 2024 East Antarctic winter heatwave. **a** 500 hPa geopotential height anomalies (shadings; unit: gpm) during July 24 to August 9, 2024. **b**, **c** Same as (**a**), but for 200 hPa and 10 hPa geopotential height anomalies, respectively. **d** Scatterplot between regional-mean T2m anomalies and 10 hPa geopotential height anomalies over Dronning Maud Land during July 24 to August 9 from 1979 to 2024. Blue dots indicate the original data, and red dots the linearly detrended ones. The year 2024 is marked by pentagrams. The linear regression line, its 95% uncertainty intervals, and the Pearson correlation coefficients (r) are also shown. **e** Daily evolution of 10-hPa zonal wind anomalies averaged over 45°–90°S (blue) and 10-hPa air temperature anomalies averaged over polar cap (60°–90°S) (red) during July–August 2024. The hatched light blue area indicates the study period. **f** Time–height evolution of standardized geopotential height anomalies (shadings) and air temperature anomalies (contours; from −4 to 4 by 1; solid for positive and dashed for negative, and zero contours are omitted) averaged over Dronning Maud Land during July–August 2024. **g** Same as **f**, but for standardized daily SAM index.

During the heatwave, the Antarctic circulation regime featured a high-pressure system over western EA and circumpolar low-pressure anomalies over the Southern Ocean, indicative of a negative SAM (Fig. 2a–c). This high-pressure system extends from the stratosphere to the surface, implying a strong troposphere–stratosphere coupling (Fig. 2d and Supplementary Fig. 4). From the beginning of July 2024, two successive SSW events substantially increased mid-stratospheric temperatures (Fig. 2e), leading to a weakened and displaced polar vortex (Supplementary Fig. 5). The resulting stratospheric high-pressure anomalies propagated downward, sustaining the pronounced negative phase of the SAM and the associated positive geopotential height anomalies into early August (Fig. 2f–g). The significant negative correlation between the SAM index and Tx17d anomalies over DML further substantiates the critical role of the SAM (Supplementary Fig. 6b, e), whereby weaker (stronger) westerlies associated with negative (positive) SAM phases enhance (suppress) the advection of warm air masses from lower latitudes. In contrast, no significant link with ENSO was detected (Supplementary Fig. 6c,d f), while Tx17d anomalies correlated positively with global mean surface temperature (GMST) (Supplementary Fig. 6a, b).

### Physical processes behind the 2024 winter heatwave

**Fig. 3:**
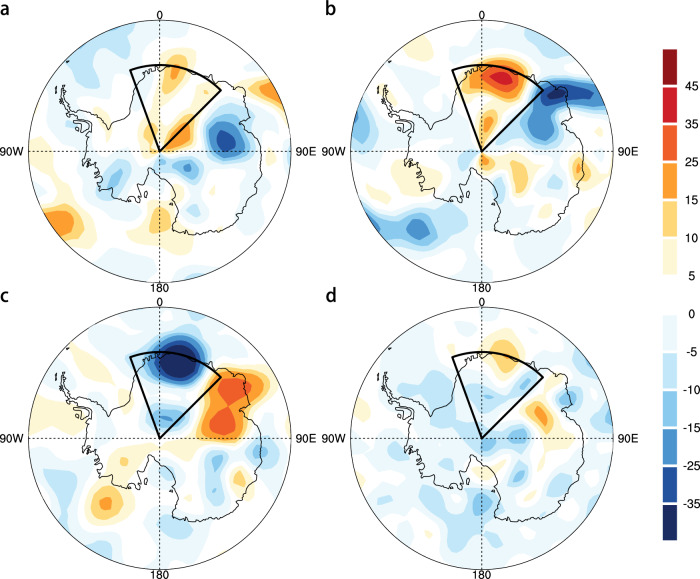
Physical processes behind the 2024 East Antarctic winter heatwave. **a** The vertically integrated zonal temperature advection anomalies (unit: K day^–^^1^) from 750 to 500 hPa during the heatwave onset period (24 to 30 July). The black box indicates the Dronning Maud Land (20°W–45°E, 90–70°S). **b** Same as **a**, but for meridional temperature advection anomalies (unit: K day^–^^1^). **c** Same as **a**, but for adiabatic warming anomalies (unit: K day^–^^1^). **d** Same as **a**, but for diabatic heating anomalies (unit: K day^-1^).

To further investigate the physical processes driving the onset and intensification of the heatwave, we apply the temperature tendency equation integrated over the mid-troposphere during the heatwave onset period (see Methods). The decomposition confirms that anomalous meridional temperature advection, linked to poleward heat transport under a blocking high over western EA, was the primary contributor (Fig. 3b). Anomalous zonal temperature advection provided a secondary contribution (Fig. 3a), while adiabatic cooling associated with coastal mechanical lift and diabatic cooling over inland DML partly offset the warming (Fig. 3c–d). Additionally, enhanced meridional moisture transport associated with the blocking high increased lower-tropospheric humidity and cloud cover over DML, strengthening downward longwave radiation (Supplementary Fig. 7). This water vapor–cloud–radiation feedback likely reinforced the surface warming associated with the heatwave^51,52^.

**Fig. 4:**
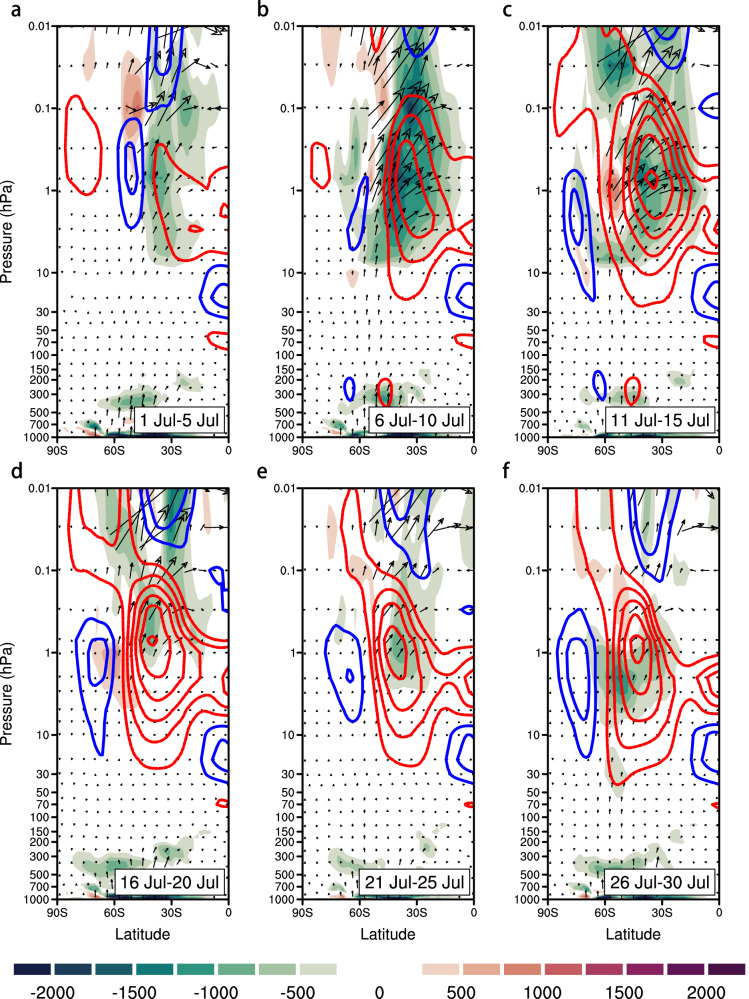
Dynamic origins underlying the 2024 polar vortex weakening. Latitude–height distribution of pentad mean EP flux anomalies of planetary waves (vectors) and their divergence (shadings; negative values indicate convergence), and zonal-mean zonal wind (contours) during 2024 (**a**) July 1 to July 5, (**b**) July 6 to July 10, (**c**) July 11 to July 15, (**d**) July 16 to July 20, (**e**) July 21 to July 25, and (**f**) July 26 to July 31. Blue (red) contours indicate positive (negative) zonal wind anomalies.

The weakening of the stratospheric polar vortex and the occurrence of early-season SSW events are relatively rare in the Southern Hemisphere, motivating a further investigation of their dynamical origins. To this end, we examine anomalous planetary wave activity during July 2024, preceding the DML heatwave. In early July (1–5 July), anomalous upward Eliassen–Palm (EP) fluxes signaled strong planetary wave propagation into the mid-stratosphere, coinciding with reduced zonal winds and preconditioning the vortex for weakening (Fig. 4a). Wave activity intensified during 6–10 July, with pronounced EP flux convergence and momentum deposition that decelerated the circumpolar westerlies (Fig. 4b). By mid-July, persistent EP flux convergence and anomalous easterlies propagated downward, further weakening the stratospheric polar vortex and preconditioning the troposphere for the subsequent heatwave (Fig. 4c). As the wave forcing intensified, a pronounced negative anomaly in the zonal-mean eddy heat flux ($\bar{v{\prime} T{\prime} }$) (see Methods) was observed at 10 hPa over 45°S–75°S (Supplementary Fig. 8). This anomaly exceeded four standard deviations and was dominated by the zonal wavenumber-1 (WN1) component, indicating the critical role of large-scale, low-frequency planetary waves in driving the vortex weakening and the early-season SSW. Although the vortex gradually recovered later in July, residual easterly anomalies in the lower stratosphere prolonged tropospheric impacts for about two weeks, implying the sustained role of downward coupling.

To explore the source of the upward propagating wave fluxes, we examine the horizontal propagation of Rossby waves prior to and during the event^53^. To visualize the Rossby wave propagation, we analyze pentad-mean 200 hPa velocity potential and stream function anomalies from July to early August 2024 (Supplementary Fig. 9). In early July, significant negative velocity potential anomalies developed east of Australia, indicating enhanced convection and upper-level divergence. This convective forcing generated poleward-propagating Rossby waves, which became more organized into a coherent wave train across the Southern Hemisphere mid-latitudes in the following pentads^54,55^. This Rossby wave propagation appears to have influenced the key blocking ridge along the EA coast. Several episodes of enhanced poleward wave propagation from South America and the subtropical Atlantic likely strengthened the ridge, connecting back to the hemispheric-scale Rossby wave generated in early July. These waves may have acted to amplify mid-latitude jet disturbances, enhance upward planetary wave propagation, and potentially contribute to the weakening of the polar vortex. The detailed mechanisms linking the convectively forced Rossby wave train to polar vortex weakening, however, require further investigation and are beyond the scope of this study.

### Storyline attribution

**Fig. 5:**
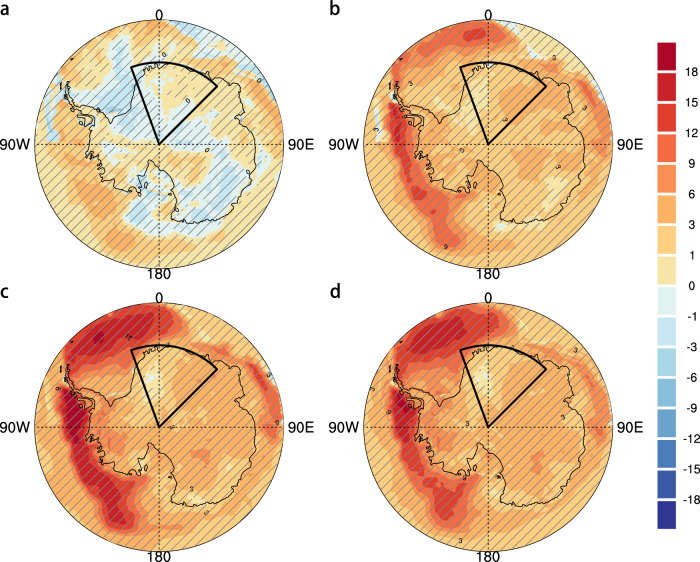
Storyline attribution of 2024 East Antarctic heatwave. **a** Difference of T2m anomalies (shadings; unit: °C) simulated during July 24 to August 9, 2024, between *Hist-WRF* and *Past-WRF* simulations. **b** Same as **a**, but between *SSP2-4.5-WRF* and *Past-WRF* simulations. **c** Same as **a**, but between *SSP5-8.5-WRF* and *Past-WRF* simulations. **d** Same as **a**, but between *SSP5-8.5-WRF* and *Hist-WRF* simulations. Hatchings indicate grid points where the difference between the two fields is statistically significant at the 90% confidence level.

To isolate the anthropogenic thermodynamic contribution to the 2024 DML winter heatwave, we first apply an event-based storyline approach by conducting a suite of simulations using the Weather Research and Forecasting (WRF) model (Table 1). Each simulation is nudged to the same observed large-scale circulation fields derived from ERA5 across all vertical levels, thereby preserving the synoptic dynamical conditions of the 2024 event (see Methods). The *Hist-WRF* simulation reproduces the observed peak warming (not shown), and analysis of the temperature tendency equation further confirms that horizontal temperature advection played a key role in driving the 2024 winter heatwave (Supplementary Fig. 10). The *Past-WRF* simulation, representing a counterfactual climate without anthropogenic influence, is constructed within the pseudo–global warming (PGW) framework^56^, in which the observed boundary conditions are adjusted to remove the estimated anthropogenic climate signal (see Methods). By retaining the observed circulation, this configuration allows us to isolate the thermodynamic contribution of anthropogenic warming while maintaining the event’s synoptic context. The results indicate a slightly weaker temperature response in the *Past-WRF* simulation, with a regional-mean anomaly over DML approximately 0.3 °C lower than that in the *Hist-WRF* simulation (Fig. 5a). Although the large-scale thermal forcing applied in WRF imposes an approximately 1 °C cooler background state in the *Past-WRF* experiment across most of Antarctica (Supplementary Fig. 11a), the resulting local temperature contrast during the heatwave is smaller. This reduction in regional warming in *Hist-WRF* is possibly due to the simulation producing weak low-pressure anomalies over western DML, despite the upper-level circulation being nudged to ERA5, which promotes enhanced cold air advection from the Antarctic interior toward the Weddell Sea (not shown).

**Table 1:** Summary of models used in the three attribution methods for the 2024 East Antarctic heatwave

Framework	Model	Model type	Configuration	Resolution	Role in analysis
Storyline	WRF	Regional	Nudged to ERA5; lateral & surface boundaries updated every 3 h	27 km	Isolate the thermodynamic contribution of anthropogenic climate change by assessing how the intensity of the 2024 event would differ under different climate states, given the same circulation.
	CMIP6 multi-model ensemble	Global	Multi-model mean Δ applied to WRF boundaries following the PGW design	1° × 1° regridded	
Circulation analogue	ERA5	Reanalysis	Observed 1981–2000 and 2001–2020	0.25° × 0.25°	Assess how anthropogenic climate change modifies event intensity under comparable dynamical conditions by comparing 2024-like events across different climate states.
	CMIP6 multi-model ensemble	Global	*Historical-CMIP6, HisNat-CMIP6*, and future projections under *SSP2-4.5* & *SSP5-8.5*	1° × 1° regridded	
Probabilistic event attribution	HadGEM3-A-N216 large ensemble	Global (Atmosphere-only)	*Hist-HadGEM3 (model evaluation), HisExt-2024* & *HisNatExt-2024*	0.56° × 0.83°	Quantify changes in the probability and magnitude of 2024-like events under anthropogenic forcing by sampling the full range of dynamically possible states.
	CMIP6 multi-model ensemble	Global	*Historical-CMIP6, HisNat-CMIP6*, and future projections under *SSP2-4.5* & *SSP5-8.5*	1° × 1° regridded	

Future projections suggest that, under the same dynamical setup, the 2024-like heatwaves would intensify by an additional 2.3 °C under the medium-emission *SSP2-4.5-WRF* simulation and 2.9 °C under the high-emission *SSP5-8.5-WRF* simulation by the end of the 21st century relative to the pre-industrial period (Fig. 5b–c). These PGW experiments characterize the projected warming as a conditional thermodynamic response to prescribed circulation patterns (i.e., if the DML heatwave were to occur under a warmer climate scenario, how would its intensity change?), rather than as a direct projection of future climate states. A plausible amplification pathway contributing to the surface temperature increase is the sea ice–albedo feedback. Warmer ocean temperatures can suppress winter sea ice formation, leading to reduced surface albedo and enhanced absorption of radiative heat, which further amplifies ocean warming. Reduced winter sea ice in the southern Atlantic Ocean, as evident in the *SSP5-8.5* simulation (Supplementary Fig. 11f), contributes to lower surface albedo and increased absorption of solar radiation, enhancing oceanic heat storage. Recent studies also show that anomalously low sea ice extent increases upward ocean heat fluxes and turbulent energy exchanges, supplying heat and moisture to the atmosphere and intensifying cyclonic activity^57^. These processes can precondition the atmosphere for enhanced meridional heat and moisture transport and surface warming under favorable synoptic configurations^58^. Thus, the even stronger warming projected for the future, relative to historical simulations, may also reflect the non-linear regional temperature responses arising from these secondary and cascading thermodynamic processes (Fig. 5d).

### Circulation analogue

**Fig. 6:**
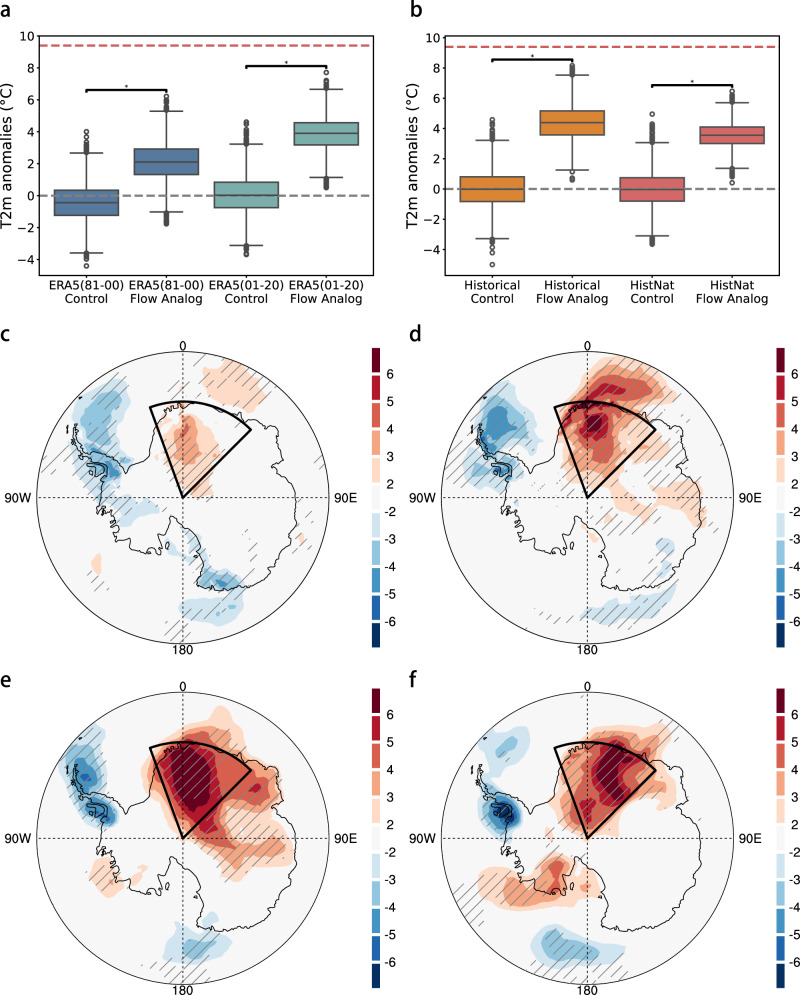
The role of the atmospheric blocking in the 2024 East Antarctic heatwave. **a** Distribution of T2m anomalies (unit: °C) on circulation analogue days compared to randomly selected control days for ERA5 reanalysis during 1981–2000 (blue) and 2001–2020 (green). Each boxplot shows the interquartile range (25th–75th percentiles), with the central line denoting the median. The horizontal red dashed line represents the observed T2m anomaly of 2024 event derived from ERA5. Statistical significance of the differences between circulation analogue and control distributions is assessed using two-sided Mann–Whitney U tests, with asterisks (*) indicating *p*-values < 0.05. **b** Same as **a**, but for *Historical-CMIP6* (orange) and *HisNat-CMIP6* (red) simulations during 1979–2014. **c** Spatial distribution of T2m anomalies (unit: °C) on flow analogue days for ERA5 reanalysis during 1981–2000. **d**–**f** Same as **c**, but for ERA5 during 2001–2020, *Historical-CMIP6* and *HisNat-CMIP6* simulations during 1979–2014, respectively. Hatchings indicate grid points where more than 90% of best-matched analogs show anomalies of the same sign.

To complement the storyline approach which focuses on the specific event, here we use the circulation analogue approach to investigate the thermodynamic contribution to the same class of heatwave events driven by a similar dynamical setup (Table 1). Leveraging the circulation analogue approach (see Methods) and ERA5 reanalysis for two specific periods (i.e., 1981–2000 and 2001–2020), we identify historical analogues for the 2024 winter event with similar eddy geopotential height anomalies at 500 hPa, representing synoptic tropospheric circulation, and then at 10 hPa, reflecting stratospheric conditions linked to SSW events, thereby ensuring dynamical consistency across both layers (Supplementary Fig. 12). These analogue days exhibit substantially higher T2m anomalies across the DML region than randomly chosen dates (*p* < 0.05), indicating the important role of atmospheric blocking in driving the 2024 winter heatwave (Fig. 6a). Compared with 1981–2000, the median T2m anomaly associated with these analogue days increases from 2.2 °C (95% uncertainty intervals [UIs]: 0.2–4.1 °C) to 3.9 °C (95% UIs: 2.2–5.5 °C) during 2001–2020, implying a median warming increment of 1.7 °C (95% UIs: −0.8–4.3 °C) under comparable circulation conditions. Here, the 95% UIs are derived from 1000 bootstrap resamples with replacement, with the relatively wide intervals primarily reflecting pronounced internal climate variability over Antarctica. Spatially, the analogue T2m pattern closely resembles the observed structure, characterized by a pronounced dipole with warming over East Antarctica and cooling over the Antarctic Peninsula (Fig. 6c, d). The DML warming signal is further amplified in the later period relative to the earlier baseline.

Following a three-step model evaluation procedure (see Methods), we select four better performing models from the Coupled Model Intercomparison Project Phase 6 (CMIP6) (ACCESS-CM2, ACCESS-ESM1-5, CNRM-CM6-1, and GFDL-ESM4) for subsequent attribution analysis. These coupled models, together with the atmosphere-only HadGEM3-A-N216 simulation, reproduce both the DML temperature variability (Supplementary Figs. 13–14) and the observed troposphere–stratosphere coupling (Supplementary Fig. 15), providing a credible representation of the mechanisms underlying the 2024 winter heatwave. Applying the same analogue selection process to the selected CMIP6 ensembles further supports the observational result (Supplementary Fig. 12). The DML regional mean T2m anomalies increase substantially in the *Historical-CMIP6* simulations under analogous circulation days compared to randomly sampled days (Fig. 6b, e). For these analogue days, the median T2m anomaly across DML reaches 4.4 °C (95% UIs: 2.5–6.2 °C), in stark contrast to the –0.01 °C median observed for randomly selected days (*p* < 0.05), indicating that the large-scale atmospheric circulation alone accounts for 47% (95% UIs: 27–66%) of the observed 2024 DML temperature anomaly. A further comparison with *HisNat-CMIP6* simulations under analogous circulation conditions suggests that anthropogenic thermodynamic forcing contributes an additional warming of about 0.8 °C (95% UIs: −1.5–3.0 °C) under 2024-like dynamic circulation (Fig. 6b, e, f).

To assess whether circulation patterns analogous to the 2024 peak heatwave circulation have undergone systematic changes under ongoing climate warming, we further examine the temporal evolution of their occurrence frequency in both reanalysis and *Historical-CMIP6* simulations (see Methods). Across a range of pattern correlation thresholds, neither ERA5 nor *Historical-CMIP6* simulations exhibit a robust or statistically significant long-term trend in the frequency of these 2024-like circulation analogues over the satellite era (Supplementary Fig. 16). It indicates that the increasing severity of the event may primarily arise from thermodynamic amplification acting on broadly stationary circulation regimes. Moreover, DML T2m anomalies under similar dynamic conditions will be further enhanced in response to increased future warming (Supplementary Fig. 17). By the end of the 21st century, these anomalies are projected to increase by 2.5 °C (95% UIs: −0.1–5.1 °C) under *SSP2-4.5* and by 7.1 °C (95% UIs: 4.7–9.5 °C) under *SSP5-8.5* relative to the present-day period. This suggests an increasing thermodynamic amplification of heatwaves associated with troposphere–stratosphere coupled blocking in a warming climate, even in the absence of significant dynamical changes.

### Probabilistic event attribution

**Fig. 7:**
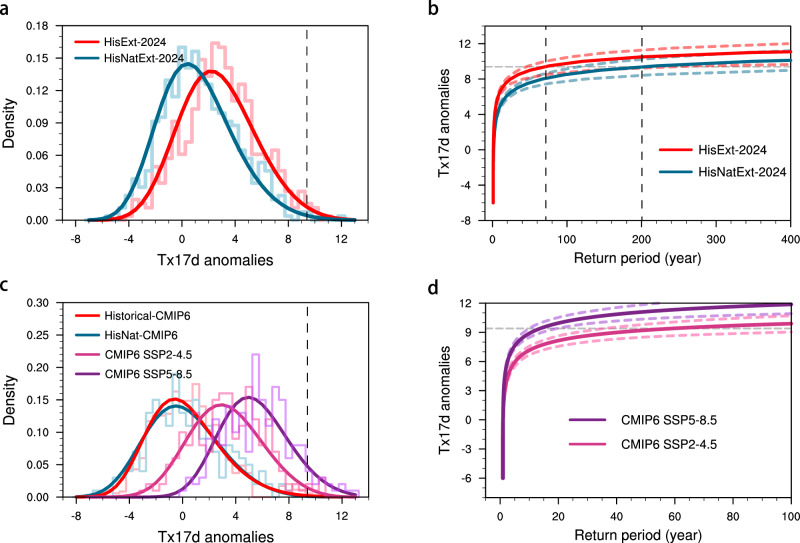
Probabilistic attribution of 2024 East Antarctic heatwave. **a** PDFs fitted using the GEV distribution (solid lines) and raw histograms (translucent lines) of Tx17d anomalies for *HisExt-2024* (red) and *HisNatExt-2024* (blue). The dashed black line marks the threshold of the observed 2024 event. **b** Return periods of Tx17d anomalies (solid lines) for *HisExt-2024* (red) and *HisNatExt-2024* (blue), with corresponding 5–95% uncertainty ranges shown as dashed lines. The horizontal dashed gray line indicates the threshold of the observed 2024 event. The vertical dashed black lines indicate the corresponding return periods. **c** Same as **a**, but for *Historical-CMIP6* (red) and *HisNat-CMIP6* (blue) during 2001–2020, and *SSP2-4.5* (pink) and *SSP5-8.5* (purple) during 2081–2100. **d** Same as **b**, but for *SSP2-4.5* (pink) and *SSP5-8.5* (purple) simulations.

Previous sections quantified the thermodynamic contribution of anthropogenic forcing to the 2024 winter heatwave under a fixed dynamical setup, and to the broader class of heatwave events occurring under similar dynamical conditions. Here, we complement the circulation-conditioned approaches with the unconditioned probabilistic analysis to assess how anthropogenic forcings alter the likelihood and magnitude of 2024-like DML heatwaves across large ensembles, independent of specific dynamical setups (Table 1). Using the atmosphere-only HadGEM3-A-N216 large ensemble, we compare simulations for 2024 forced by observed sea surface temperature (SST) and sea ice concentration (SIC) conditions (*HisExt-2024*) with counterfactual simulations using the same SST and SIC but excluding anthropogenic forcings (*HisNatExt-2024*) (see Methods). Anthropogenic forcing shifts the probability density functions (PDFs) of Tx17 d anomalies toward warmer values (Fig. 7a), increasing the likelihood of 2024-like events from 0.7% (95% UIs: 0.3–1.3%) (P_NAT_) to 1.9% (95% UIs: 1.0–2.8%) (P_ALL_). This corresponds to a probability ratio (PR) of 2.9 (95% UIs: 1.2–6.8) and a reduction in return period from ~200 to ~70 years (Fig. 7b). Here, PR quantifies the change in event likelihood attributable to anthropogenic forcing and is defined as P_ALL_ divided by P_NAT_ (see Methods). The magnitude shift, defined as the temperature difference between the factual and counterfactual simulations at the observed return period, is 1.1 °C (95% UIs: 0.1–2.0 °C), indicating that anthropogenic warming not only increases the frequency but also amplifies the intensity of 2024-like heatwaves.

Next, we analyze coupled CMIP6 simulations, in which the atmosphere, ocean, and sea ice evolve interactively, to assess the robustness of the probabilistic attribution results derived from the atmosphere-only HadGEM3-A-N216 ensemble. Compared to their natural counterparts, *Historical-CMIP6* simulations including all anthropogenic forcings also exhibit a shift toward higher temperatures in the PDF (Fig. 7c), although the shift appears less pronounced than in the HadGEM3-A-N216 results, possibly constrained by a relatively small ensemble size. Specifically, the probability of 2024-like heatwaves increases from 0.2% (95% UIs: 0.0–0.4%) in *HisNat-CMIP6* to 0.4% (95% UIs: 0.2–0.6%) in *Historical-CMIP6*, corresponding to a PR of 2.2 (95% UIs: 0.6–27.6). The anthropogenic forcing is estimated to contribute a magnitude shift of approximately 0.6 °C (95% UIs: −0.7–1.7 °C). Future CMIP6 projections under the *SSP2-4.5* and *SSP5-8.5* pathways indicate a more pronounced anthropogenic influence (Fig. 7c). By the end of 21st century, the likelihood of 2024-like heatwaves is projected to increase to 1.9% (95% UIs: 1.0–3.0%) under *SSP2-4.5* and 9.5% (95% UIs: 6.6–12.1%) under *SSP5-8.5* scenario, corresponding to PRs of 5.9 (95% UIs: 2.6–14.3) and 26.4 (95% UIs: 14.0–74.6), respectively. The return periods are projected to further decline to around 50 and 10 years under the *SSP2-4.5* and *SSP5-8.5* scenarios, respectively (Fig. 7d). The associated magnitude shifts are projected to reach 1.9 °C (95% UIs: 0.8–3.0 °C) and 3.8 °C (95% UIs: 2.9–4.5 °C), respectively, relative to the present-day climate.

The attribution and projection results from the multi-model, multi-method framework are synthesized to provide an integrated assessment (Table 2). Despite methodological differences and variations in conditioning, all approaches consistently indicate that anthropogenic warming played a non-trivial role in the 2024 and 2024-like heatwaves. Across present-day attribution experiments, the synthesis yields a mean magnitude shift of approximately 0.7 °C (based on the unweighted average of individual best estimates across frameworks) and a PR of 2.6 (based on the unweighted mean of probabilistic estimates from HadGEM3-A-N216 and CMIP6) relative to the natural-forcing baseline. These values should be interpreted as indicative central estimates rather than formally combined metrics, as the averaging does not account for inter-model dependence, structural uncertainty, or differences in experimental design. Overall, the cross-framework consistency strengthens confidence in the attribution results, demonstrating a detectable contribution of anthropogenic warming to both the thermodynamic amplification and the increased likelihood of the 2024 DML heatwave and analogous events under comparable circulation conditions.

**Table 2:** Summary of anthropogenic contributions to the 2024 East Antarctic heatwave across attribution frameworks

Framework	Conditioning	Climate state	Magnitude shift (°C)	Probability ratio (PR)
Storyline (PGW)	High (Conditioned on the observed large-scale circulation)	Hist (present-day) vs Past	0.3	—
		*SSP2-4.5* (end of 21st century) vs Past	2.3	—
		*SSP5-8.5* (end of 21st century) vs Past	2.9	—
Circulation analogue	Medium (Conditioned on circulation patterns similar to those observed during the event)	2001–2020 vs 1981–2000 (ERA5)	1.7 (−0.8–4.3)	—
		Historical vs HisNat (present-day)	0.8 (−1.5–3.0)	—
		*SSP2-4.5* (end of 21st century) vs historical (present-day)	2.5 (−0.1–5.1)	—
		*SSP5-8.5* (end of 21st century) vs historical (present-day)	7.1 (4.7–9.5)	—
Probabilistic event attribution	Low (Thermodynamic and dynamic effects)	HadGEM3-A-N216 HisExt-2024 vs HisNatExt-2024 (present-day)	1.1 (0.1–2.0)	2.9 (1.2–6.8)
		CMIP6 Historical vs HisNat (present-day)	0.6 (−0.7–1.7)	2.2 (0.6–27.6)
		*SSP2-4.5* (End of 21st century) vs Historical (present-day)	1.9 (0.8–3.0)	5.9 (2.6–14.3)
		*SSP5-8.5* (End of 21st century) vs Historical (present-day)	3.8 (2.9–4.5)	26.4 (14.0–74.6)

The 95% UIs are derived from 1000 bootstrap resamples with replacement. All estimates and associated 95% UIs are reported to one decimal place.

## Discussion

During July–August 2024, an unprecedented heatwave across DML raised regional temperatures more than 9 °C above climatology for 17 consecutive days, representing the strongest winter temperature anomaly since the start of the satellite period in 1979. Compared with the March 2022 heatwave, which stands as the strongest on record^9,10^, this event unfolded in the core of austral winter and coincided with the earliest stratospheric warming in four decades and the second-lowest sea ice extent. In this study, we identify the weakening of the Antarctic stratospheric polar vortex as the primary driver, which generated quasi-barotropic high-pressure anomalies and triggered the heatwave through enhanced meridional heat and moisture transport.

From the dynamical origin perspective, the anomalous weakening of the stratospheric polar vortex in 2024 can be possibly attributed to enhanced upward propagation of tropospheric planetary waves. Intensified tropical convection likely amplified these waves, facilitating their penetration into the stratosphere, where increased wave breaking and associated momentum deposition systematically decelerated the westerly jet. This disruption of the vortex circulation established a preconditioned state that favored the onset of the DML winter heatwave. In addition to tropical forcing, recent studies have suggested the role of anomalous Antarctic sea-ice anomalies in modulating mid-latitude planetary wave activity: reduced sea ice extent can weaken the surface baroclinicity, alter waveguide properties, and thus reinforce the upward-propagating wave flux into the stratosphere^12,59^. Taken together, these findings indicate a multi-scale coupling, linking tropical convection, Antarctic sea ice variability, and stratospheric circulation, that ultimately shapes the DML winter heat extremes. However, the specific quantification of each feedback pathway, particularly the potential interaction between Antarctic sea ice anomalies and polar vortex dynamics, remains limited by current observational and modelling constraints, and thus represents an important avenue for future investigation.

To attribute the event, we present a multi-model, multi-method framework combining (I) synoptic-nudged high-resolution regional WRF simulations, (II) HadGEM3-A-N216 large ensemble driven by 2024 observed SST and SIC, and (III) free-running CMIP6 simulations. The three methods address different facets of the attribution problem and have complementary strengths and limitations. The PEA framework, based on HadGEM3-A-N216 large ensemble and CMIP6 simulations, quantifies changes in the likelihood of 2024-like heatwave events under different forcing scenarios, integrating both thermodynamic and dynamical contributions. While this approach provides robust estimates of probabilistic attribution, this comes at the expense of resolving the specific dynamical features of the observed 2024 event. The circulation analogue approach, by conditioning on similar large-scale circulation states, enables a clearer separation of thermodynamic effects from circulation-driven dynamic variability. However, both approaches are primarily linear in nature and may not fully capture potential non-linear responses. The storyline approach is therefore employed, allowing internally generated perturbations in processes such as atmosphere–ocean heat exchange, turbulent fluxes, and cloud–radiation interactions, even under prescribed boundary field conditions^56^. This framework enables the identification of physically plausible, thermodynamically driven non-linear responses that may not be fully captured by probabilistic or circulation analogue analyses based on global climate model ensembles. Nevertheless, this method does not provide a formal probabilistic sampling of internal variability or structural model uncertainty, as it relies on a limited ensemble. The results should therefore be interpreted as circulation-conditioned sensitivity experiments rather than quantitative estimates of event probability. By combining these three complementary methodologies, we assess the robustness of the attribution results across frameworks and provide a more complete picture in which anthropogenic warming plays a non-trivial role in amplifying the 2024 DML winter heatwave. Consequently, what would have been a multi-century heatwave under natural forcing has become a centennial-scale event in the present-day climate.

In summary, the 2024 DML winter heatwave is not merely an isolated event, but a potential harbinger of more systematic Antarctic climate shifts driven by anthropogenic warming. Although the event produced record-breaking temperature anomalies, most surface temperatures remained well below the melting point. Nevertheless, with continued background warming, winter and shoulder-season heatwaves, along with increasingly frequent summer extremes, are expected to bring coastal and ice shelf regions closer to or above the melting threshold^19^. In addition, episodes of wetter snowfall can refreeze into impermeable near-surface ice layers that enhance lateral meltwater routing and favor the formation and persistence of surface melt ponds. The resulting concentration of meltwater increases hydrostatic pressure on pre-existing fractures, thereby facilitating melt-pond–driven hydrofracturing of ice shelves^7^. Reduced sea ice extent further facilitates ocean heat uptake and allows atmospheric rivers to penetrate deeper inland^21^. Together, these processes may weaken ice shelf buttressing and accelerate mass loss from outlet glaciers^60^. Beyond the cryosphere, such extremes may also disrupt Antarctic ecosystems, threatening the resilience of cold-adapted species and regional food webs^61,62^. Addressing these challenges will require targeted satellite-based cryosphere monitoring, expanded in situ observation networks, integrated biological surveys, and refined polar climate modeling, combined with strong emission reduction efforts to moderate the pace of climate change in the highly sensitive Antarctic region.

## Methods

### Station and reanalysis data

This study uses atmospheric and surface variables primarily from the fifth-generation reanalysis dataset of the European Centre for Medium-Range Weather Forecasts (ECMWF), ERA5^63^. ERA5 provides daily near-surface air temperature (T2m), precipitation, sea ice concentrations (SIC), outgoing longwave radiation (OLR), surface heat flux, specific humidity, vertical integral of eastward and northward water vapor flux, and key atmospheric circulation variables (including horizontal winds and geopotential heights). The dataset has a horizontal resolution of 0.25° × 0.25° and includes 37 pressure levels from 1000 to 1 hPa. We focus on data from 1979 onward, since satellite-based observational coverage at high southern latitudes is limited before this period^64^.

To assess whether there is consensus between different datasets, we also examine daily atmospheric variables from the Japanese Reanalysis for Three Quarters of a Century (JRA-3Q)^65^. JRA-3Q provides a horizontal resolution of 1.25° × 1.25° and includes 45 vertical levels extending from 1000 to 1 hPa. To evaluate the reliability of reanalysis dataset, we incorporate in situ observations from Antarctic weather stations, with T2m records extending up to 31st August 2024 (Supplementary Table 1). These station-based measurements are obtained from the Research Data for Environmental and Antarctic Research (READER) dataset, which undergoes rigorous quality control procedures, including outlier removal and homogenization to account for instrument changes and bias correction.

### CMIP6 simulations

To attribute and investigate the projected changes of 2024-like heatwave events, we use simulations from 14 coupled global climate models archived in the Detection and Attribution Model Intercomparison Project (DAMIP) of the Coupled Model Intercomparison Project Phase 6 (CMIP6)^66^. All available ensemble members for each model are incorporated to increase the sample size (Supplementary Table 2).

The *Historical-CMIP6* experiment involves simulations that incorporate both anthropogenic influences (such as greenhouse gases (GHGs), anthropogenic aerosols, and land-use changes) and natural forcings (including volcanic aerosols and solar irradiance) spanning 1850 to 2014. In contrast, the *HisNat-CMIP6* experiment involves simulations that are driven solely by natural forcings. To represent the present-day climate background, we select the period 2001–2020, which captures recent climate conditions while minimizing the influence of major volcanic eruptions, such as the 1991 Mount Pinatubo event.

For long-term future projections (2015–2100), we analyze two shared socioeconomic pathway–representative concentration pathway (SSP–RCP) scenarios. *SSP2–4.5* represents an intermediate pathway in terms of socioeconomic development, land use, adaptation and mitigation strategies, and GHG emissions, resulting in radiative forcing near 4.5 W m^–^^2^ by 2100. In contrast, *SSP5–8.5* represents a high-forcing scenario associated with fossil-fuel-intensive development, limited mitigation, and continued high GHG emissions, yielding near 8.5 W m^–^^2^ radiative forcing at the end of the century. The period 2081–2100 is chosen to represent the end-of-century climate response under these scenarios.

### HadGEM3-A-N216 large ensemble

The Met Office Hadley Centre attribution system is employed as well to assess the role of anthropogenic forcing in both the probability change and magnitude shift of the 2024-like heatwave events. This system is based on the HadGEM3-A-N216 model, an atmosphere-only configuration of the Hadley Centre Global Environment Model version 3 (HadGEM3), which features a horizontal resolution of 0.56° × 0.83° (N216 spectral truncation) and 85 vertical levels^67^.

The HadGEM2-A-N216 simulations comprise three distinct simulation ensembles. Firstly, the *Hist-HadGEM3* experiment comprises 15 initial-condition ensemble members and spans the period 1960–2013. It is driven by observed sea surface temperatures (SST) and SIC, and accounts for both anthropogenic and natural forcings. Secondly, the *HisExt-2024* experiment consists of 525 members, each driven by observed 2024 SST and SIC boundary conditions. This large ensemble samples not only atmospheric internal variability but also parametric uncertainty by perturbing key values in the model’s parameterization schemes^68^. By altering the representation of processes such as convection, radiation, and cloud microphysics, these alternative parameter settings expand the range of simulated climate responses, increasing ensemble size and enhancing the robustness of attribution. Thirdly, the *HisNatExt-2024* experiment also consists of 525 members, but isolates the influence of natural forcings by excluding anthropogenic effects. It uses adjusted 2024 SST and SIC fields, modified to retain only the natural variability inherent in these boundary conditions. Specifically, observed anthropogenic trends are removed from the SST and SIC datasets, thereby preserving variability driven solely by natural climate processes.

### Storyline simulations based on pseudo-global warming approach

We construct event-based storylines of the 2024 East Antarctic winter heatwave using the Weather Research and Forecasting (WRF) model to simulate the heatwave evolution under different climatic backgrounds (Table 1). Four sets of simulations are conducted, in which large-scale atmospheric fields across all vertical levels, from the troposphere to the stratosphere, are nudged toward the ERA5 reanalysis throughout the event period, while allowing thermodynamic variables to evolve freely. This approach provides a physically consistent framework for quantifying the thermodynamic role of anthropogenic forcing in modifying the intensity of the 2024 winter heatwave. A four-day spin-up period is applied to all WRF simulations to ensure model equilibration from the initial conditions and to establish dynamically consistent surface conditions.

The *Hist-WRF* simulation represents the actual atmospheric conditions during the heatwave and is driven by ERA5 reanalysis, with lateral and surface boundary conditions updated every three hours. This setup provides a baseline state of the atmospheric circulation and thermodynamic environment of the event. To assess how the event would have occurred in a climate without anthropogenic influence, we conduct the *Past-WRF* simulation using the well-established pseudo–global warming (PGW) delta adjustment approach applied to CMIP6 historical simulations. This framework is designed to isolate the thermodynamic imprint of anthropogenic climate change on a specific circulation-driven event, rather than to reproduce the actual historical evolution of the climate system or changes in the likelihood of the associated large-scale circulation. In this method, large-scale boundary conditions from the reanalysis are adjusted by removing the model-estimated anthropogenic climate change signal. Specifically, we use the CMIP6 multi-model ensemble mean to quantify the long-term forced changes between the modern baseline (1985–2014) and the pre-industrial period (1850–1879). Fourteen variables, including temperature, horizontal winds, geopotential height, SST, and SIC, are modified by subtracting these ensemble-mean climatological differences. The resulting boundary conditions represent a pre-industrial thermodynamic background, while maintaining the same large-scale circulation and weather-sequence characteristics of the observed event.

To evaluate the potential impacts of future warming, we conduct two additional simulations based on CMIP6 SSP–RCP scenarios. *SSP2-4.5-WRF* simulation represents a moderate-emission and intermediate socio-economic future, with boundary conditions adjusted according to projected climate differences for 2070–2099 relative to 1985–2014 baseline under the *SSP2-4.5* scenario. Similarly, *SSP5-8.5-WRF* simulation reflects a high-emission, high-growth future, with boundary conditions modified according to *SSP5-8.5* projections for the same period. These experiments allow us to quantify how the intensity of the 2024 winter heatwave would be thermodynamically altered under different future climates, conditional on the observed circulation.

### Climate indices

All reanalysis and model datasets are regridded to a common 1° × 1° grid using bilinear interpolation to maintain consistency. The study region is defined as Dronning Maud Land (DML) (20°W–45°E, 90–70°S) in Antarctica (Fig. 1a), where the 2024 winter heatwave is most pronounced. All datasets (masked by study region) are converted to anomalies at each grid point relative to the baseline climatology (1981–2010). The regional-mean T2m time series is then obtained as the area-weighted average across all grid points, with each point weighted according to the surface area it represents, accounting for latitude-dependent grid spacing. Sensitivity test using alternative climatological reference period (1979–2024) is also conducted, and the results remain robust.

The Antarctic heatwave is identified when daily T2m exceeds the 90th percentile of the climatological distribution over the reference period (1981–2010) for at least three consecutive days. If two heatwave episodes are separated by no more than one non-heatwave day, they are treated as a single continuous heatwave event. The 90th percentile threshold is calculated using a 5-day running mean, which reduces daily variability and enlarges the sample for the percentile calculation by including overlapping values from adjacent days. Sensitivity analyses using 11- and 15-day windows yield consistent results. Adopting higher thresholds, such as the 95th or 99th percentile, results in shorter identified heatwave durations, but the overall spatiotemporal characteristics remain robust. To quantify the intensity of the 2024 DML heatwave, we define Tx17d as the maximum regional mean daily T2m over a 17-day period during mid-winter (July–August, JA)^69^, corresponding to the 17-day duration of the event from 24 July to 9 August. Calculating Tx17d over the entire winter season (June–August) yields consistent results.

The Southern Annular Mode (SAM) patterns are identified as the first empirical orthogonal function (EOF) of the July–August mean geopotential height anomalies^70^. These anomalies are calculated across all pressure levels from 1000 to 10 hPa, south of 20°S, for the period from 1979 to 2024. To ensure that the eigenvalues are significantly separated, the North’s test is applied to confirm the robustness of the leading EOF. The daily SAM index is calculated by projecting the spatial field of daily geopotential height anomalies at each pressure level onto the SAM pattern. This projection produces a single principal component value for each day, which serves as a temporal measure of SAM variability.

Global mean surface temperature (GMST) anomalies are computed as the annual mean area-weighted average of global T2m anomalies relative to the pre-industrial baseline (1861–1890). The El Niño–Southern Oscillation (ENSO) is represented by the Niño3.4 index, defined as SST anomalies averaged over the central eastern equatorial Pacific (120°W–170°W, 5°S–5°N) during July–August. Statistical significance in this study is determined using the two-tailed Student’s *t*-test or Mann–Whitney U test.

### Extreme value analysis

We simulate the Tx17d ($y$) distributions using the non-stationary generalized extreme value (GEV) distribution, which is governed by three parameters: location parameter ($\mu$), scale parameter ($\sigma$), and shape parameter ($\xi$). The GEV distribution is defined as follows:

1 $$G\left({y|}\mu ,\sigma ,\xi \right)=\left\{\begin{array}{l}exp\left\{-{\left[1+\xi (y-\mu )/\sigma \right]}^{-1/\xi }\right\},\xi > 0,y < \mu -\sigma /\xi \\ exp\left\{-exp\left[-(y-\mu )/\sigma \right]\right\},\xi =0\\ exp\left\{-{\left[1+\xi (y-\mu )/\sigma \right]}^{-1/\xi }\right\},\xi < 0,y > \mu -\sigma /\xi \end{array}\right.$$

We shift the GEV location parameter proportionally to changes in 4-year smoothed GMST to calculate the return period, while the scale and shape parameters are held fixed. The return period is defined as the inverse of the exceedance probability based on non-stationary GEV distribution.

### Temperature tendency equation and planetary wave diagnosis

To investigate the physical drivers of the 2024 heatwave, we use the temperature tendency equation, which can be expressed as:

2 $$\mathop{\underbrace{\partial T/\partial t}}\limits_{{TT}}=\mathop{\underbrace{-u(\partial T/\partial x)}}\limits_{{UAdv}}\mathop{\underbrace{-v(\partial T/\partial y)}}\limits_{{VAdv}}\mathop{\underbrace{-\omega (\partial \theta /\partial p){(p/{p}_{0})}^{R/{C}_{p}}}}\limits_{{Adiab}}\mathop{\underbrace{+Q/{C}_{p}}}\limits_{{Diab}}$$

where $T$ represents the air temperature, $t$ is time, $u$ is the zonal wind, $v$ is the meridional wind, $\omega$ is the vertical pressure velocity, $\theta$ is the potential temperature, $p$ is the pressure, ${p}_{0}$ is the surface pressure, $R$ is the gas constant, ${C}_{p}$ is the specific heat at constant pressure, and $Q$ is the total diabatic heating (including radiation, latent heating, and surface heat flux). The term on the left-hand side of the equation indicates the temperature tendency (*TT*), while the four terms on the right-hand side correspond to zonal temperature advection (*UAdv*), meridional temperature advection (*VAdv*), adiabatic warming (*Adiab*), and diabatic heating (*Diab*), respectively. Considering the high elevation of East Antarctic plateau, we restrict the integration of the temperature tendency equation to atmospheric levels above the surface, between 750 and 500 hPa. Extending the integration up to 200 hPa yields consistent results.

In this study, we employ the Eliassen–Palm (EP) flux to capture the vertical propagation of quasi-stationary Rossby waves, with its convergence and divergence serving as a diagnostic tool for wave–mean flow interactions in the stratosphere. The zonal mean eddy heat flux, $\overline{{v}^{{\prime} }{T}^{{\prime} }}$, averaged between 75°S and 45°S at 10 hPa, is used to indicate planetary wave energy entering the stratosphere. In this expression, the bar denotes the zonal mean, while ${v}^{{\prime} }$ and ${T}^{{\prime} }$ represent deviations of the meridional wind velocity and air temperature from their respective zonal means. Moreover, we apply Fourier transformation to decompose atmospheric fields into components with different zonal wavenumbers. This allows us to identify the roles of specific wave structures at various spatial scales in shaping the 2024 polar vortex weakening event in the Southern Hemisphere. The Rossby wave with zonal wavenumber-1 (WN1) is referred to as the planetary wave.

### Model evaluation

Prior to attribution, we conduct a three-step evaluation of HadGEM3-A-N216 and CMIP6 simulations (Supplementary Table 2) to assess their ability to reproduce key characteristics of observed East Antarctic winter temperature variability. The model evaluations are based on the first ensemble member of each model.

Firstly, we assess how well the models simulate the spatial pattern of mid-winter T2m over DML during the baseline period (1981–2010). To quantify model performance, we employ the *S-index*:

3 $$S=\frac{4(1+R)}{{(\sigma +1/\sigma )}^{2}\left(1+{R}_{0}\right)}$$

where $R$ indicates the pattern correlation coefficient between the simulated and observed T2m fields, and $\sigma$ is the ratio of their spatial standard deviations. ${R}_{0}$ is the maximum attainable correlation, assumed to be 1. Simulations with an *S-index* close to 1 are considered highly skillful, whereas lower values indicate poorer performance.

Secondly, we apply the non-parametric Kolmogorov–Smirnov (K–S) test to determine whether the simulated and observed T2m distributions originate from the same statistical distribution. If $p > 0.05$, we fail to reject the null hypothesis, indicating no significant difference between the simulated and observed distributions. Moreover, we use the interannual variability skill (${IVS}$) score to quantify the models’ ability in capturing temperature fluctuations:

4 $${IVS}={\left[\frac{ST{D}_{sim}}{ST{D}_{obs}}-\frac{ST{D}_{obs}}{ST{D}_{sim}}\right]}^{2}$$

where ${{STD}}_{{sim}}$ and ${{STD}}_{{obs}}$ denote the interannual standard deviations of the simulated and observed regional-mean T2m, respectively. Lower *IVS* scores (close to 0) indicate better model performance in replicating observed interannual variability. To further evaluate the models’ capability in simulating extreme winter temperature events, we perform GEV parameter estimation for each model using regional mean DML Tx17d anomalies. The location, scale, and shape parameters are estimated via maximum likelihood, and 95% uncertainty intervals are calculated using bootstrap resampling. Models are considered skillful if their estimated GEV parameters lie within the 95% uncertainty intervals of the corresponding ERA5 and JRA-3Q GEV parameters.

Thirdly, we evaluate the representation of troposphere–stratosphere coupling in the simulations by examining the relationship between regional mean T2m and geopotential height anomalies at 500 hPa, 200 hPa, and 10 hPa. This assessment determines whether the models can reliably capture the large-scale atmospheric dynamics responsible for the 2024 heatwave, focusing on the downward propagation of stratospheric anomalies into the troposphere.

The results suggest that both HadGEM3-A-N216 and CMIP6 simulations exhibit good performance in reproducing the climatological DML temperature pattern and matching observed anomaly distributions (K–S test, *p* > 0.05) (Supplementary Table 2; Supplementary Figs. 13, 14). Moreover, based on the GEV parameter estimates, most models are identified as capable of capturing the heat extremes as ERA5, except for two CMIP6 models (CESM2 and IPSL-CM6A-LR), which are excluded from further attribution (Supplementary Table 3). However, when evaluating their representation of large-scale atmospheric dynamics, most CMIP6 models fail to reproduce the observed troposphere–stratosphere coupling, as evidenced by weak correlations between T2m anomalies and 10 hPa geopotential height anomalies (Supplementary Fig. 15). Only four CMIP6 models (ACCESS-CM2, ACCESS-ESM1-5, CNRM-CM6-1, and GFDL-ESM4) and HadGEM3-A-N216 surpass the 95% significance threshold, indicating a robust representation of the stratospheric influence on surface temperatures in these models. Based on these assessments, we select four better performing CMIP6 models (ACCESS-CM2, ACCESS-ESM1-5, CNRM-CM6-1, and GFDL-ESM4) and HadGEM3-A-N216 in the formal attribution analysis.

### Circulation analogue

Anomalous circulation days during the 2024 winter heatwave (24 July–9 August) are identified by computing daily 500 hPa and 10 hPa eddy geopotential height anomalies (90°S–60°S and 0°–360°E) relative to the 1981–2010 climatology based on ERA5 dataset. Here, the eddy geopotential height is defined as the deviation from the zonal mean at each latitude to exclude the effect of global warming on the atmospheric column. For each target day, a pool of candidate analogs is drawn from other years within a ± 30-day window around the calendar date. Analog selection proceeds in two stages: an initial ranking of the 500 hPa anomaly fields, from which the 100 most similar days (by minimum Euclidean distance) are retained, followed by a refinement using 10 hPa eddy geopotential height anomalies to choose the 20 best-matched analogs. We also measure similarity using the pattern correlation coefficient, and the two similarity metrics yield qualitatively consistent results.

To reconstruct the surface temperature response conditioned on circulation, we perform a Monte Carlo sampling of the analog set. On each of 5000 iterations, one of the 20 analog days is randomly selected for each of the 17 anomalous circulation dates, and its sequence of daily T2m anomalies, over the full duration of the heatwave, is concatenated and averaged to yield a single event mean anomaly. This procedure produces a probability distribution of T2m anomalies that reflects only the observed synoptic-scale circulation. A control distribution is generated by drawing 17-day sequences at random (without regard to circulation) from the T2m record, using the same number of iterations, to represent the unconditioned variability. By comparing the conditional and unconditional distributions, we isolate and quantify the contribution of the anomalous circulation pattern to 2024-like heatwaves.

While the circulation analogue method isolates the thermodynamic contribution under fixed circulation conditions, it does not quantify potential changes in the frequency of such circulation patterns under climate warming. To address this limitation, we further analyze the temporal evolution of circulation patterns analogous to the peak circulation of 2024 event. The peak circulation day of the 2024 winter heatwave is defined as the day with the largest spatially averaged DML T2m anomaly. Using this day as a reference, analogous circulation patterns are identified for each year based on eddy geopotential height anomalies. For the ERA5 reanalysis, daily analogs are determined based on the pattern correlation coefficient of eddy geopotential height anomalies at both 500 hPa and 10 hPa during 1979–2023. For each year, the number of days exceeding a prescribed similarity threshold is counted, yielding the time series of analogue occurrence frequency. The same analysis is applied to *Historical-CMIP6* simulations during 1980–2014 as well. This approach enables the assessment of changes in the occurrence of 2024-like circulation patterns and their evolution in both observations and climate model simulations, providing complementary insights into the dynamical contribution of climate change.

### Probability ratio and magnitude shift

To quantify the influence of anthropogenic forcing on the occurrence probability and intensity of 2024-like heatwave events, we compute both the Probability Ratio (PR) and the magnitude shift metric, respectively. The PR is defined as the ratio between the probability of the event occurring in ‘factual’ scenarios (with anthropogenic forcings) and in ‘counterfactual’ scenarios (where anthropogenic forcings have been eliminated). A PR value greater than 1 indicates that anthropogenic climate change has increased the probability of the event, while a value less than 1 indicates a reduced probability. If the confidence interval of PR includes unity, the anthropogenic signal cannot be considered statistically detectable. The corresponding 95% uncertainty interval for the PR is estimated using bootstrapping, performing 1000 resampling iterations with replacement^71^. The magnitude shift quantifies the change in event intensity attributable to anthropogenic forcing. It is computed as the temperature difference between the factual and counterfactual scenarios at the return period of the 2024-like event^72^. The corresponding 95% uncertainty interval is estimated using the bootstrap method as well.

## Supplementary information

Supplementary Information

## Data Availability

All data supporting the findings of this study are openly accessible. ERA5 datasets could be retrieved at https://cds.climate.copernicus.eu/cdsapp#!/dataset/reanalysis-era5-pressure-levels-monthly-means?tab=overview. JRA3Q datasets could be obtained from https://rda.ucar.edu/datasets/d640000/dataaccess/#. The CMIP6 model outputs can be accessed at the ESGF portal (https://esgf-node.llnl.gov/search/cmip6/). The outputs of HadGEM3-A-N216 large ensemble simulations are available from the Met Office at https://data.ceda.ac.uk/badc/eucleia/data/EUCLEIA/output/MOHC/HadGEM3-A-N216. The WRF model version used for the PGW experiments is available from https://gitlabext.wsl.ch/atmospheric-models/CRYOWRF and the package used to generate the PGW deltas is available at https://github.com/sergigonzalezh/PGWERA5WRF. The daily READER datasets can be obtained from https://legacy.bas.ac.uk/met/READER/.

## Code availability

All the codes for analyses and diagnostics are available from the corresponding author upon reasonable request.
