# Supplementary material for: Unprecedented 2024 East Antarctic winter heatwave driven by polar vortex weakening and amplified by anthropogenic warming
Source: NPJ Clim Atmos Sci. 2026 Apr 1;9(1):122. doi: 10.1038/s41612-026-01392-x (PMC13229936; doi:10.1038/s41612-026-01392-x)
Supplement: Supplementary file 1 — Supplementary Information [file 41612_2026_1392_MOESM1_ESM.pdf]

Supporting information for

Unprecedented 2024 East Antarctic Heatwave Driven by Polar Vortex Weakening and Amplified by Anthropogenic Warming

Haosu Tang[1*], Sihan Li[1], Julie M. Jones[1], Sergi González-Herrero[2], Andrew Orr[3], Friederike E.L. Otto[4], James A. Screen[5], Kyle R. Clem[6], Deniz Bozkurt[7], Jennifer L. Catto[5], Charlie C. Suitters[5], Michelle L. Maclennan[3], Yiming Sun[8]

[1]School of Geography and Planning, University of Sheffield, Sheffield, UK

[2]WSL Institute for the Snow and Avalanche Research (SLF), Davos, Switzerland

[3]British Antarctic Survey, Cambridge, UK

[4]Grantham Institute, Imperial College, London, UK

[5]Department of Mathematics and Statistics, University of Exeter, Exeter, UK

[6]School of Geography, Environment and Earth Sciences, Victoria University of Wellington, Wellington, New Zealand

[7]Departamento de Meteorología, Universidad de Valparaíso, Valparaíso, Chile

[8]School of Electrical and Electronic Engineering, University of Sheffield, Sheffield, UK

Contents:

1. Supplementary Tables 1–3
2. Supplementary Figs. 1–17

Supplementary Table 1. Details of Dronning Maud Land weather stations used in this study.

Station	Latitude	Longitude	Elevation (m)	Data period
Dome Fuji	77.31°S	39.70°E	3810	1995/02/08–2024/08/31
Amundsen Scott	90°S	–	2835	1957/01/09–2024/08/31
Syowa	69°S	39.57°E	21	1957/02/09–2024/08/31
Mizuho	70.70°S	44.29°E	2260	2000/10/07–2024/08/31
Relay station	74.02°S	43.05°E	3353	1995/02/01–2024/08/31

Supplementary Table 2. Details of 14 CMIP6 models and HadGEM3-A-N216 used in this study, and their corresponding *S-index*, *IVS* score and P value of the K–S test. The selected models are highlighted with asterisks (*).

Model	Institute	Atmospheric Resolution (latitude × longitude)	*S-index*	*IVS* score	P values
ACCESS-CM2*	CSIRO–BOM/Australia	1.25°×1.875°	0.941	0.089	0.998
ACCESS-ESM1-5*	CSIRO–BOM/Australia	1.24°×1.875°	0.958	0.022	0.952
BCC-CSM2-MR	BCC-CMA/China	1.125°×1.125°	0.992	0.189	0.952
CanESM5	CCCma/Canada	2.81°×2.81°	0.992	0.197	0.952
CESM2	NSF-DOE-NCAR/US	0.94°×1.25°	0.993	0.020	0.998
CNRM-CM6-1*	CNRM-CERFACS/France	1.4°×1.4°	0.973	0.022	0.998
FGOALS-g3	LASG-CESS/China	2.25°×2°	0.974	0.306	0.798
GFDL-CM4	NOAA-GFDL/US	1°×1.25°	0.976	0.021	0.952
GFDL-ESM4*	NOAA-GFDL/US	1°×1.25°	0.981	0.071	0.998
HadGEM3-GC31-LL	MOHC/UK	1.25°×1.875°	0.991	0.025	0.952
IPSL-CM6A-LR	IPSL/France	1.26°×2.5°	0.962	0.053	0.952
MIROC6	MIROC/Japan	1.4°×1.4°	0.952	0.022	0.952
MRI-ESM2-0	MRI/Japan	1.125°×1.125°	0.978	0.009	0.998
NorESM2-LM	NCC-NMI/Norway	1.875°×2.5°	0.987	0.001	0.952
HadGEM3-A-N216*	MOHC/UK	0.56° × 0.83°	0.997	0.003	0.998

Supplementary Table 3. GEV parameter estimates for winter Tx17d anomalies in Dronning Maud Land. The shape (ξ), location (μ), and scale (σ) parameters are estimated for each model and reanalysis. Values in parentheses indicate 95% uncertainty intervals obtained via bootstrap resampling.

**Model**	**Shape Parameter (ξ)**	**Location Parameter (μ)**	**Scale Parameter (σ)**
ACCESS-CM2	0.282 (-0.040, 0.803)	-0.715 (-1.488, 0.290)	2.004 (1.416, 2.713)
ACCESS-ESM1-5	0.326 (0.056, 0.702)	-0.623 (-1.352, 0.280)	1.917 (1.243, 2.542)
BCC-CSM2-MR	0.293 (-0.216, 0.707)	-0.489 (-1.106, 0.161)	1.391 (0.861, 1.745)
CanESM5	0.281 (-0.192, 1.116)	-0.494 (-1.087, 0.238)	1.366 (0.881, 2.439)
CESM2	0.521 (0.268, 1.108)	-0.380 (-1.103, 0.312)	1.770 (1.277, 2.831)
CNRM-CM6-1	0.524 (0.196, 1.131)	-0.437 (-1.347, 0.419)	2.058 (1.451, 3.550)
FGOALS-g3	0.215 (-0.062, 1.072)	-0.509 (-1.060, 0.144)	1.248 (0.900, 1.833)
GFDL-CM4	0.091 (-0.213, 0.578)	-0.704 (-1.277, 0.138)	1.392 (0.977, 1.960)
GFDL-ESM4	0.292 (-0.063, 0.752)	-0.695 (-1.644, 0.251)	1.960 (1.444, 2.655)
HadGEM3-GC31-LL	0.355 (0.038, 0.810)	-0.512 (-1.171, 0.131)	1.667 (1.087, 2.211)
IPSL-CM6A-LR	0.530 (0.321, 1.067)	-0.448 (-1.225, 0.274)	2.124 (1.379, 2.924)
MIROC6	0.375 (-0.262, 1.155)	-0.578 (-1.492, 0.428)	1.915 (1.099, 3.347)
MRI-ESM2-0	0.113 (-0.192, 0.418)	-0.705 (-1.295, 0.001)	1.461 (1.083, 1.837)
NorESM2-LM	0.262 (-0.081, 0.518)	-0.636 (-1.241, 0.084)	1.735 (1.020, 2.253)
HadGEM3-A-N216	0.207 (-0.129, 0.570)	-0.695 (-1.504, 0.078)	1.691 (1.233, 2.102)
ERA5	0.241 (-0.067, 0.699)	-0.650 (-1.359, 0.185)	1.679 (1.267, 2.100)
JRA-3Q	0.215 (-0.040, 0.613)	-0.678 (-1.308, 0.069)	1.685 (1.169, 2.087)

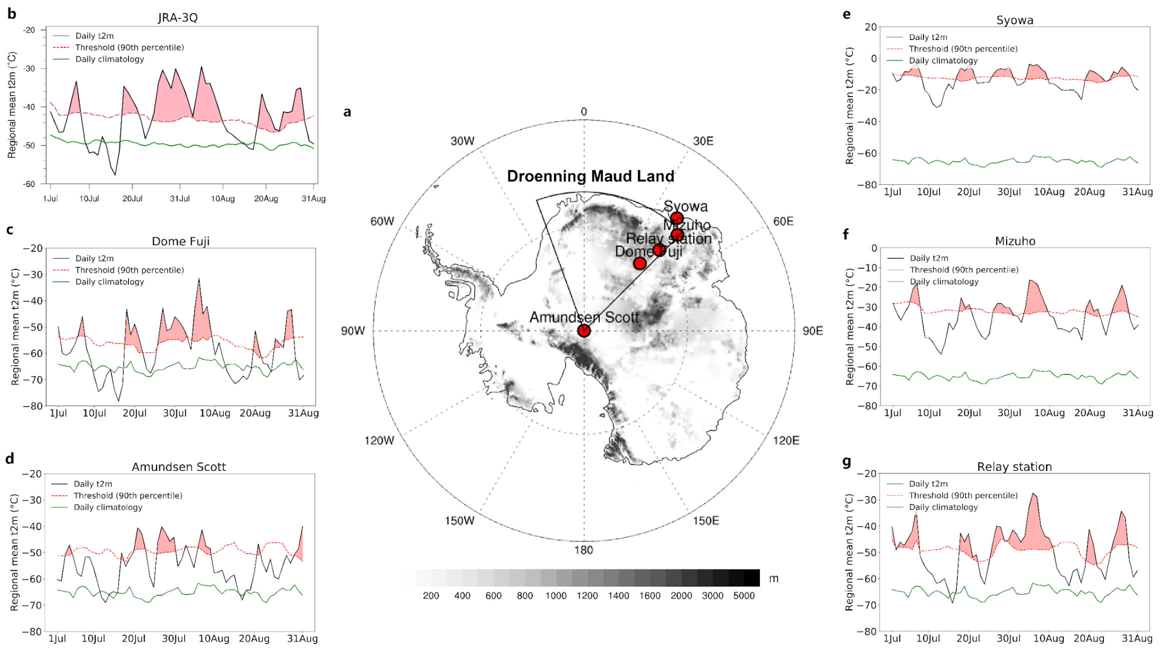

Supplementary Fig. 1 | Temperature evolution in JRA-3Q dataset and individual weather stations during mid-winter 2024. (a) Locations of Dronning Maud Land weather stations used in this study (red dots) superimposed on elevation (shadings; unit: m). The black box indicates the Dronning Maud Land. (b) Same as Figure 1c, but for JRA-3Q dataset. (c–g) Same as Figure 1c, but in Dome Fuji, Amundsen Scott, Syowa, Mizuho, and Relay station, respectively.

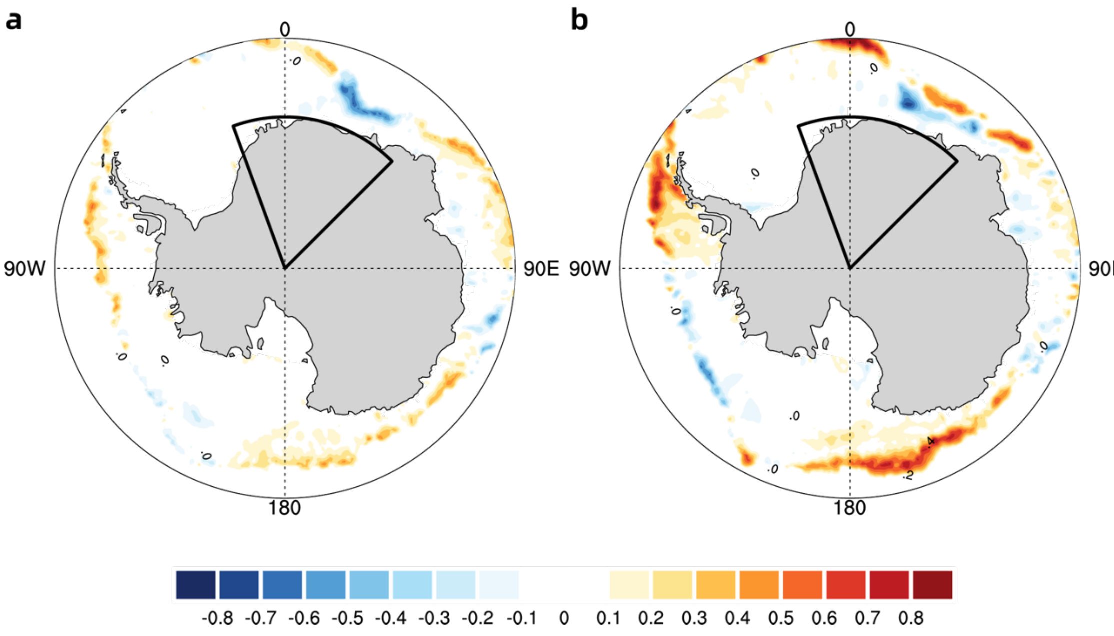

Supplementary Fig. 2 | (a) Difference in sea ice concentration between 24 July and 30 July 2024. The black box indicates the Dronning Maud Land. (b) Same as (a), but between 9 August and 24 July 2024.

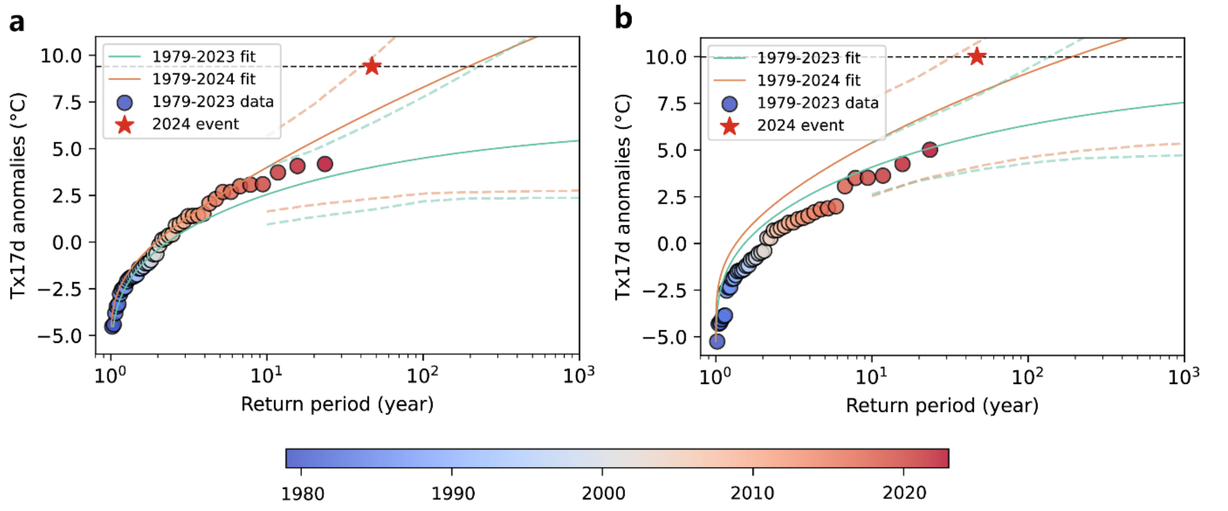

Supplementary Fig. 3 | Return period for the 2024 Dronning Maud Land heatwave. (a) Empirical (dots) and non-stationary GEV-fitted return period of Tx17d anomalies (solid curves) and the corresponding 5%–95% uncertainty ranges from 1979 to 2024 for ERA5 dataset. The GEV-fitted return period using data only from 1979 to 2023 (dashed curves) and the corresponding 5%–95% uncertainty ranges are also shown. The year 2024 is marked by pentagram. (b) Same as (a), but for JRA-3Q dataset.

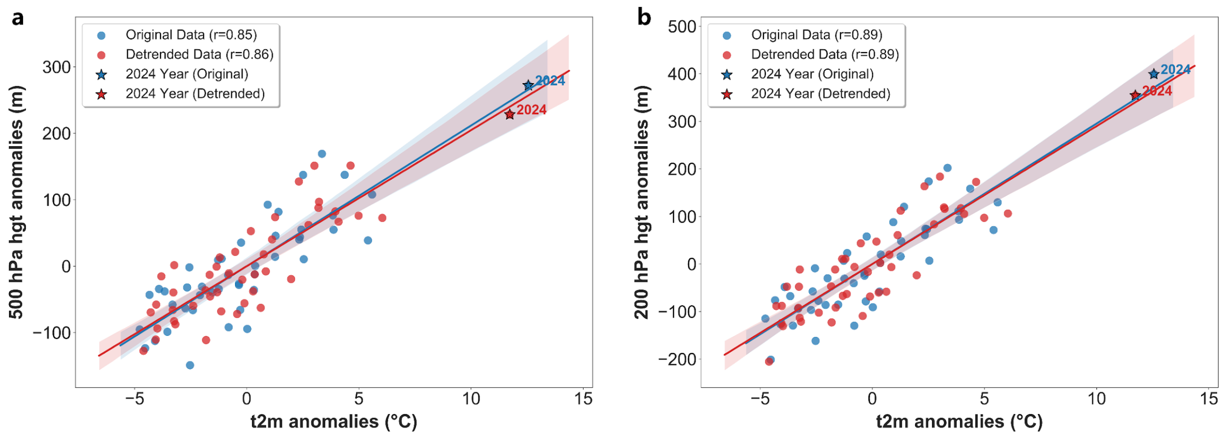

Supplementary Fig. 4 | Same as Fig. 2d, but for (a) 500 hPa and (b) 200 hPa geopotential height anomalies.

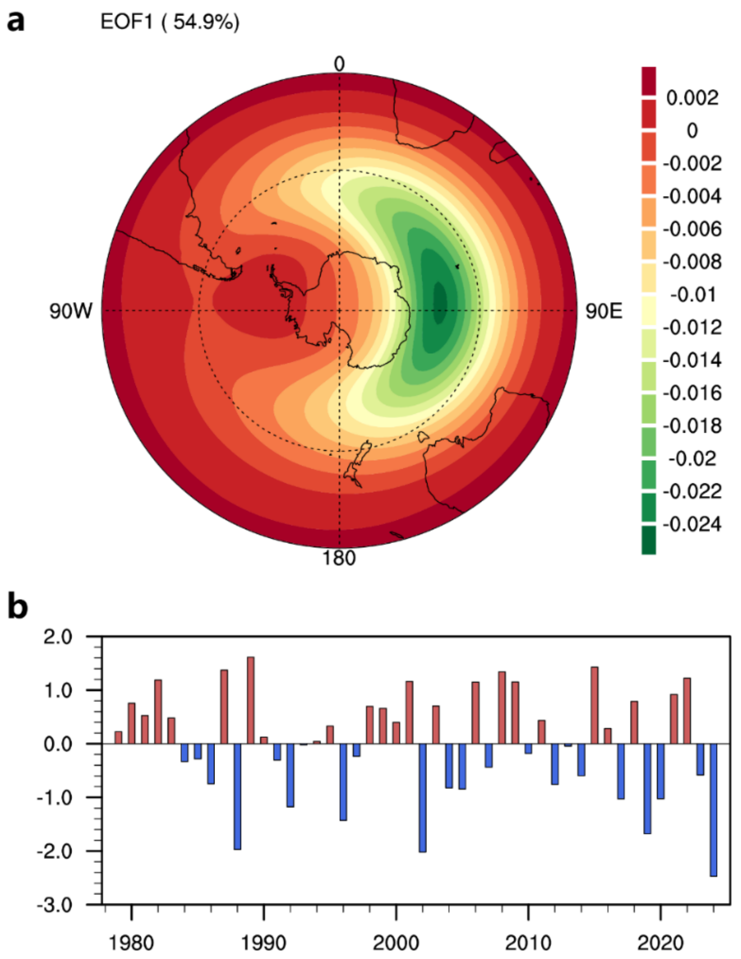

Supplementary Fig. 5 | Leading mode of the stratospheric polar vortex during July–August and the associated principal component time series. (a) First leading EOF mode of the 10 hPa daily geopotential height anomaly southward of 20°S from July to August. (b) The standardized principal component associated with the first leading EOF mode.

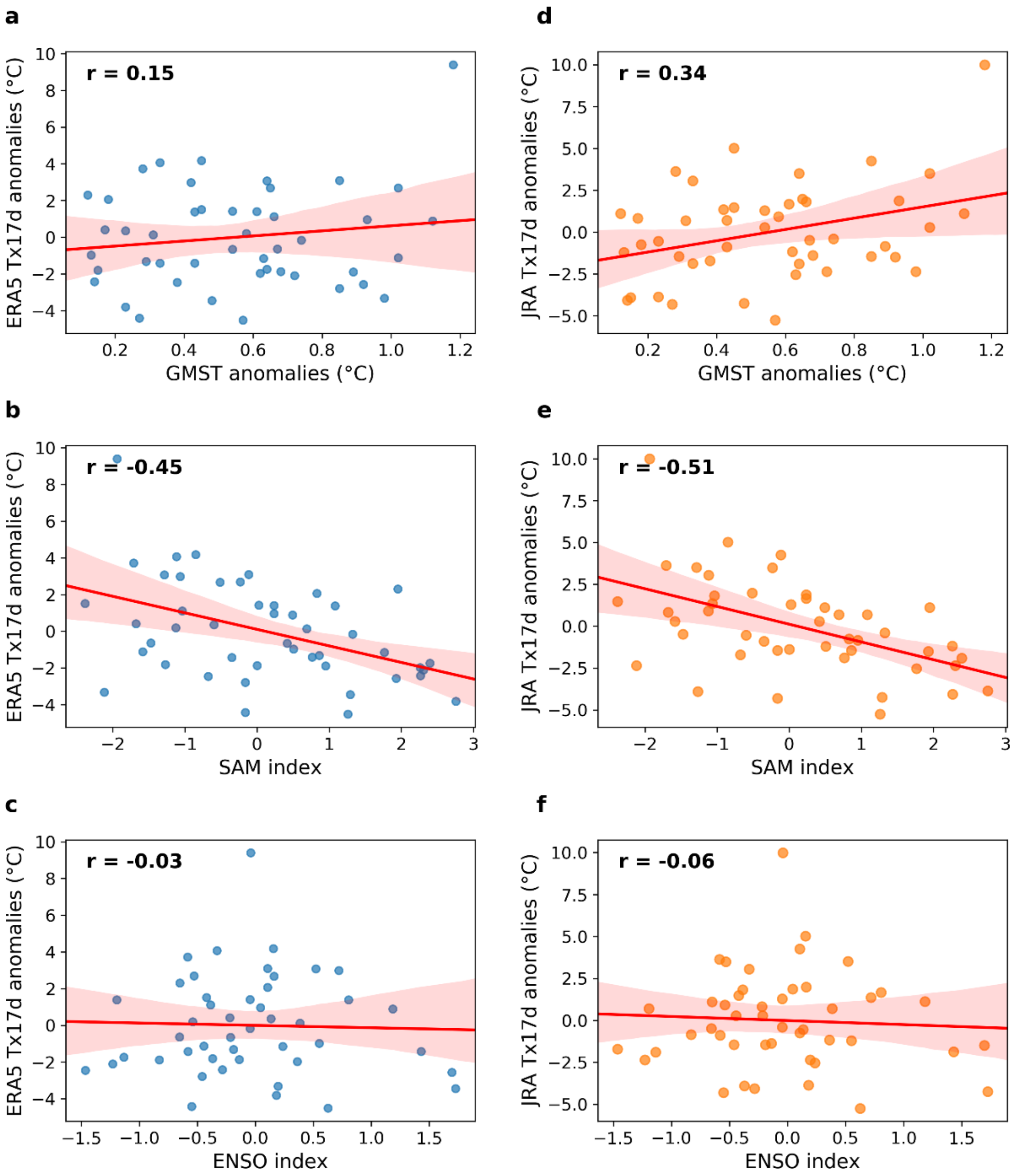

Supplementary Fig. 6 | Response of heat extremes to global climate modes. (a) Response of Tx17d anomalies (unit: °C) over Dronning Maud Land derived from ERA5 dataset for July–August to 4-year smoothed GMST changes from 1979 to 2024. The linear regression line, its 95% uncertainty intervals, and the Pearson correlation coefficients (r) are also shown. (b) Same as (a), but for SAM index. (c) Same as (a), but for ENSO index. (d–f) Same as (a–c), but for JRA-3Q dataset.

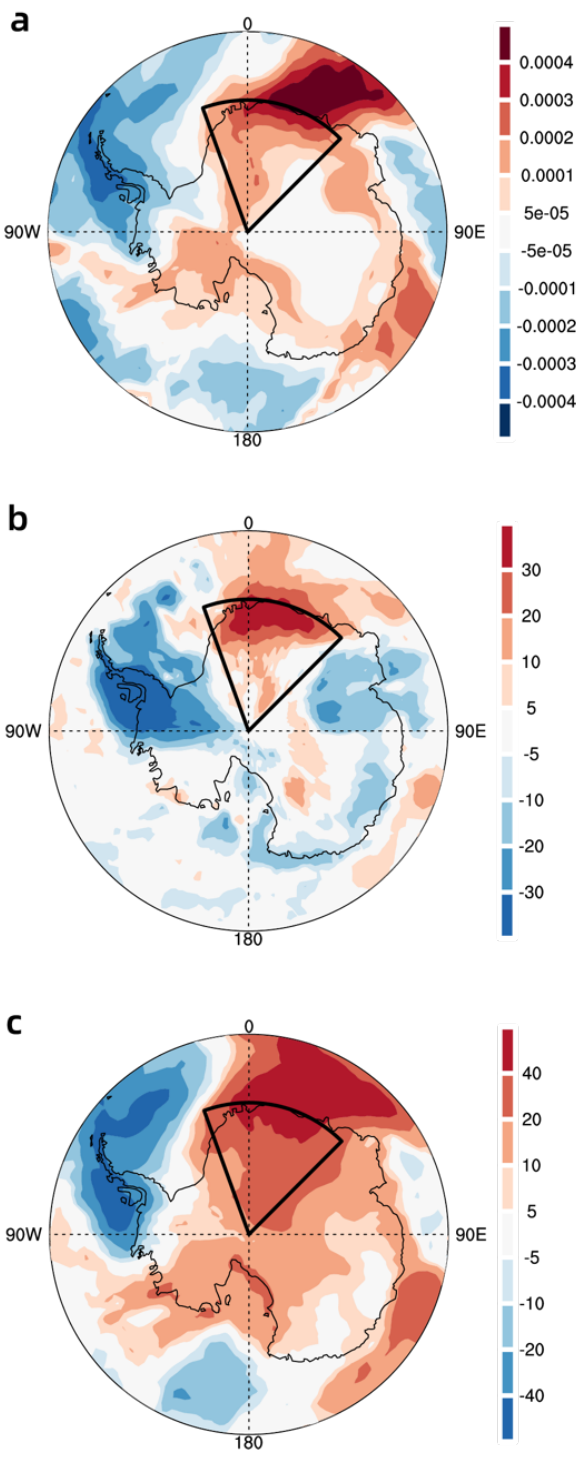

Supplementary Fig. 7 | Physical mechanism of local water vapor–cloud–radiation feedback. (a) 700 hPa specific humidity anomalies (unit: g kg^{-1}). (b) Total cloud cover anomalies (unit: %). (c) The downward longwave radiation anomalies (unit: W m^{-2}) during the study period. The black box indicates the Dronning Maud Land.

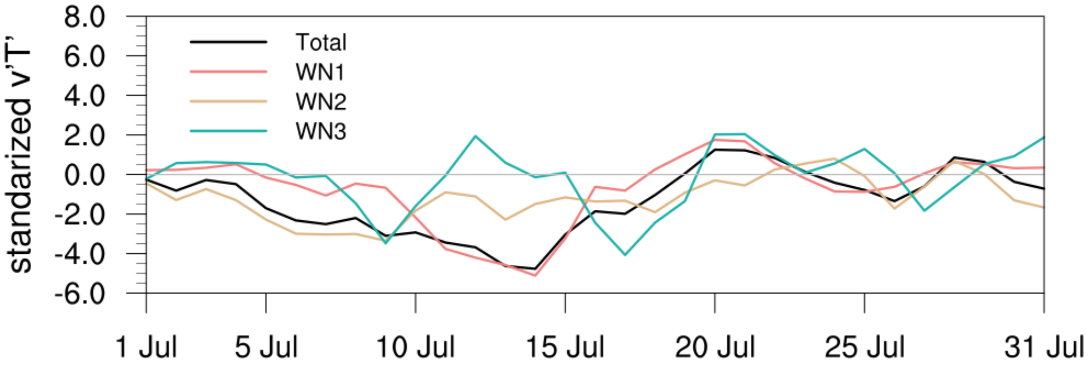

Supplementary Fig. 8 | Daily evolution of the standardized zonal mean eddy heat flux $\overline{v'T'}$ (black line), and its WN1 (red line), WN2 (brown line), and WN3 (green line) components averaged over 45°S–75°S at 10 hPa from 1 July to 31 July 2024.

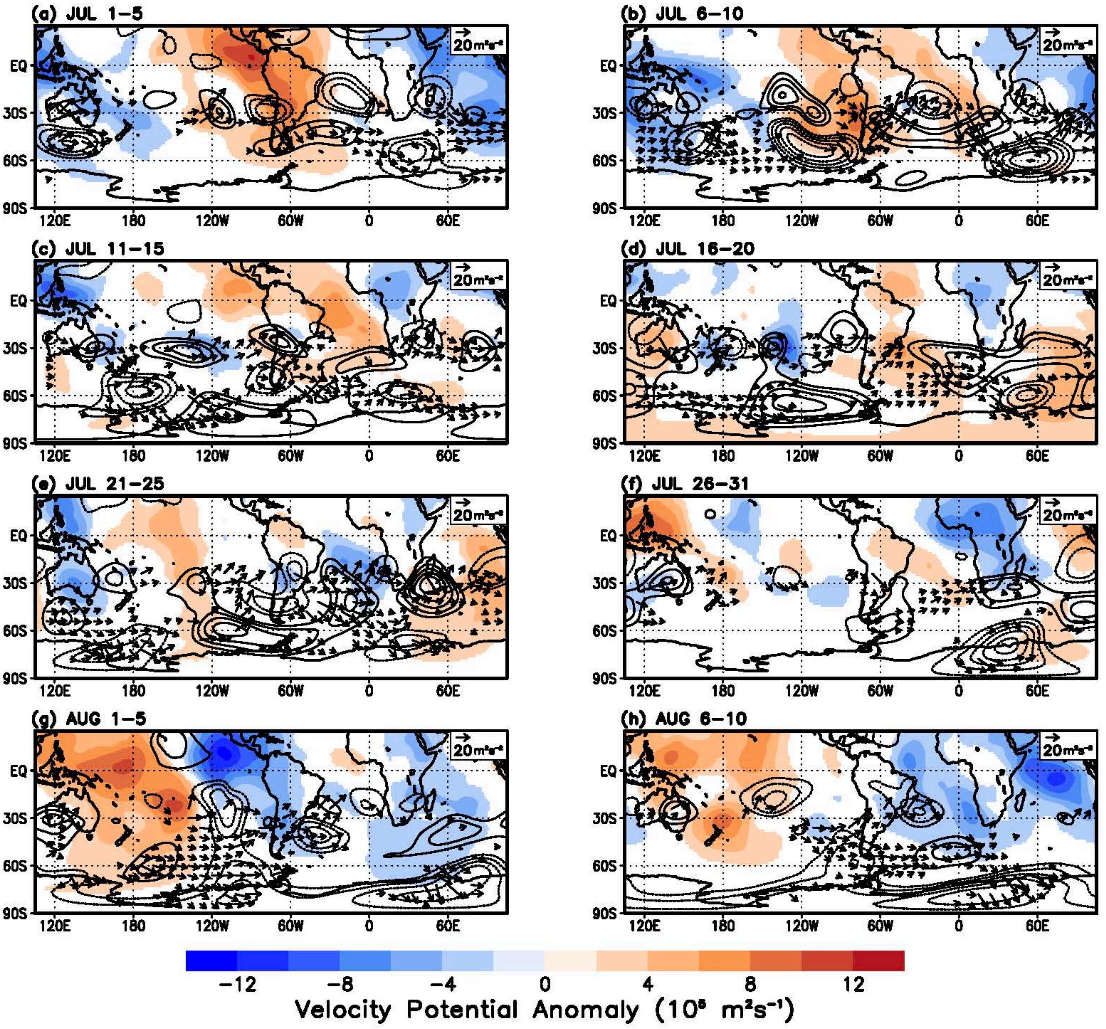

Supplementary Fig. 9 | Pentad mean 200 hPa velocity potential anomalies (shadings), stream function anomalies (contours), and the corresponding wave activity fluxes during July to early August 2024.

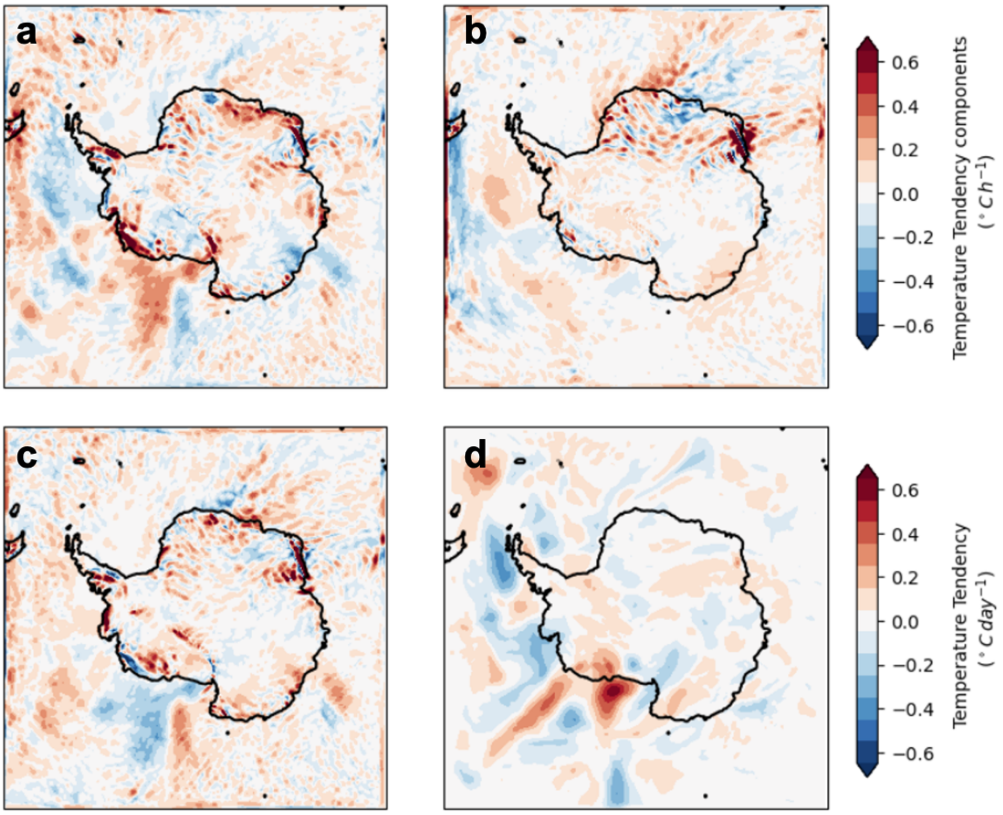

Supplementary Fig. 10 | (a) Horizontal temperature advection from the *Hist-WRF* simulation at model level σ = 0.25 during the heatwave onset period (24–30 July). (b–d) Same as (a), but for adiabatic warming, diabatic heating, and temperature tendency, respectively.

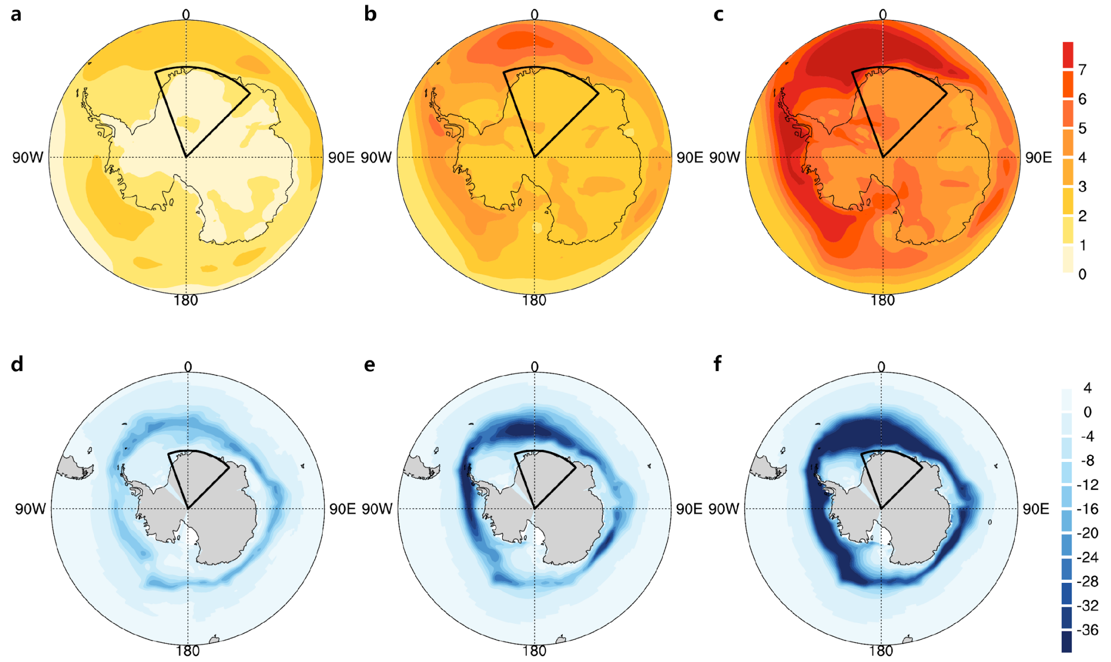

Supplementary Fig. 11 | (a) Difference of surface 2-m air temperature (shadings; unit: °C) during July to August between *Hist-WRF* and *Past-WRF* simulations. The black box indicates the Dronning Maud Land. (b) Same as (a), but between *SSP2-4.5-WRF* and *Hist-WRF* simulations. (c) Same as (a), but between *SSP5-8.5-WRF* and *Hist-WRF* simulations. (d–f) Same as (a–c), but for sea ice concertation (shadings; unit: %).

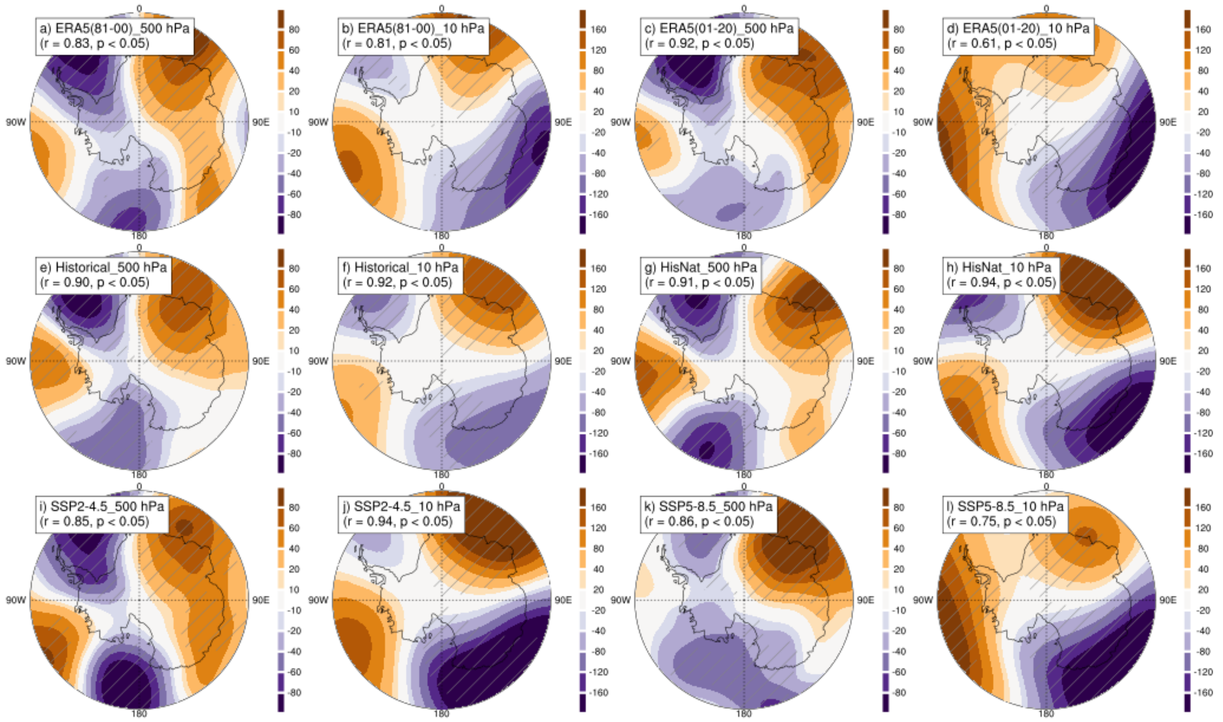

Supplementary Fig. 12 | (a, c, e, g, i, k) Spatial distribution of 500 hPa eddy geopotential height anomalies (unit: gpm; zonal mean has been subtracted to eliminate the influence of the global atmospheric column expansion induced by climate warming) on flow analogue days for six scenarios: (a) ERA5 reanalysis during 1981–2000, (c) ERA5 reanalysis during 2001–2020, (e) *Historical-CMIP6* simulations during 1979–2014, (g) *HisNat-CMIP6* simulations during 1979–2014, (i) *SSP2-4.5* projections during 2080–2100, and (k) *SSP5-8.5* projections during 2080–2100. Spatial correlation coefficients with the observed field (90°S–60°S and 0°–360°E) and corresponding p-values are also indicated. (b, d, f, h, j, l) Same as (a, c, e, g, i, k), but for 10 hPa eddy geopotential height anomalies (unit: gpm). Hatchings indicate grid points where more than 90% of best-matched analogs show anomalies of the same sign.

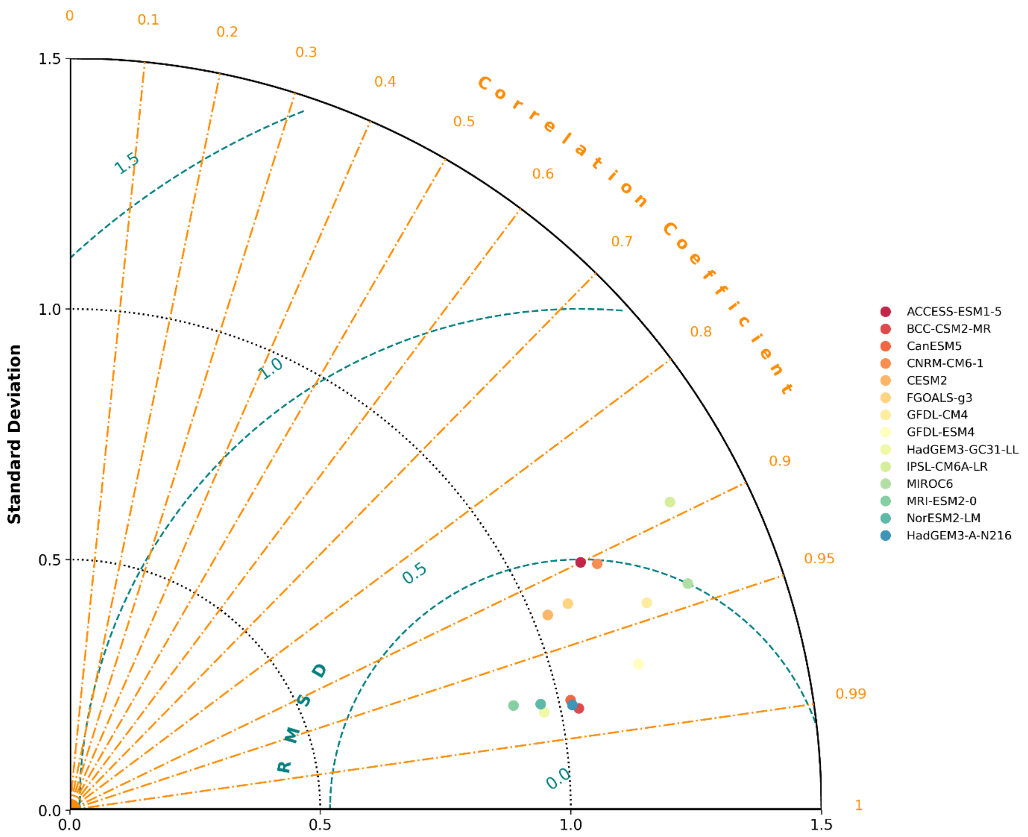

Supplementary Fig. 13 | Taylor diagram of 14 CMIP6 models and HadGEM3-A-N216 for simulating the climatological mean of t2m averaged over the study period and study region. The black circular arcs indicate the standard deviation, while the green circular arcs represent the root mean square error. The orange radial lines denote the spatial correlation coefficient.

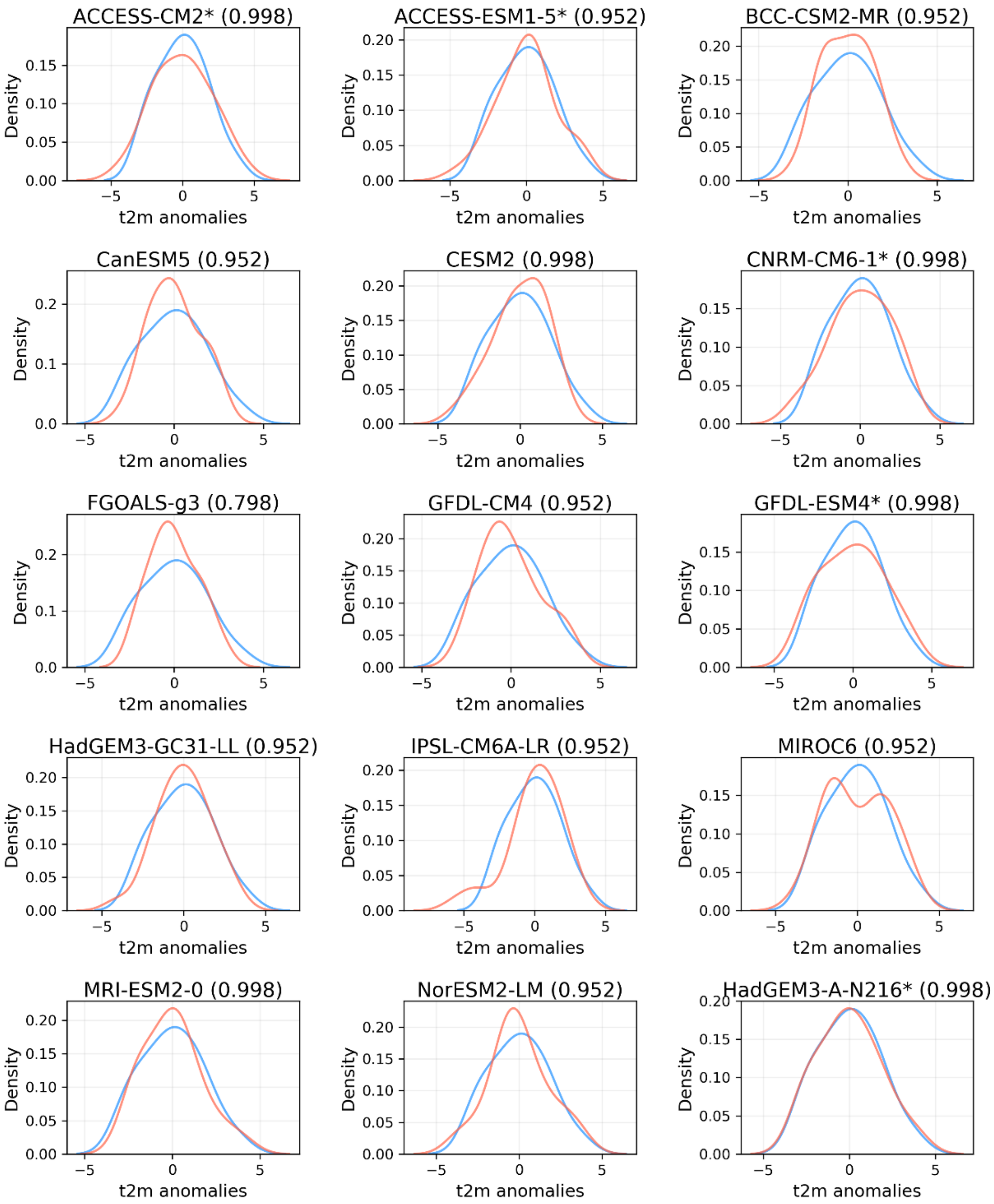

Supplementary Fig. 14 | PDFs of the regional mean t2m averaged over the study period and study region for 14 CMIP6 models and HadGEM3-A-N216 in comparison with ERA5 reanalysis. The red lines represent the ERA5, while the blue lines correspond to the respective models. The values in parentheses indicate the significance level of the K–S two-sample test between the ERA5 and the respective models. The selected models are highlighted with asterisks (*).

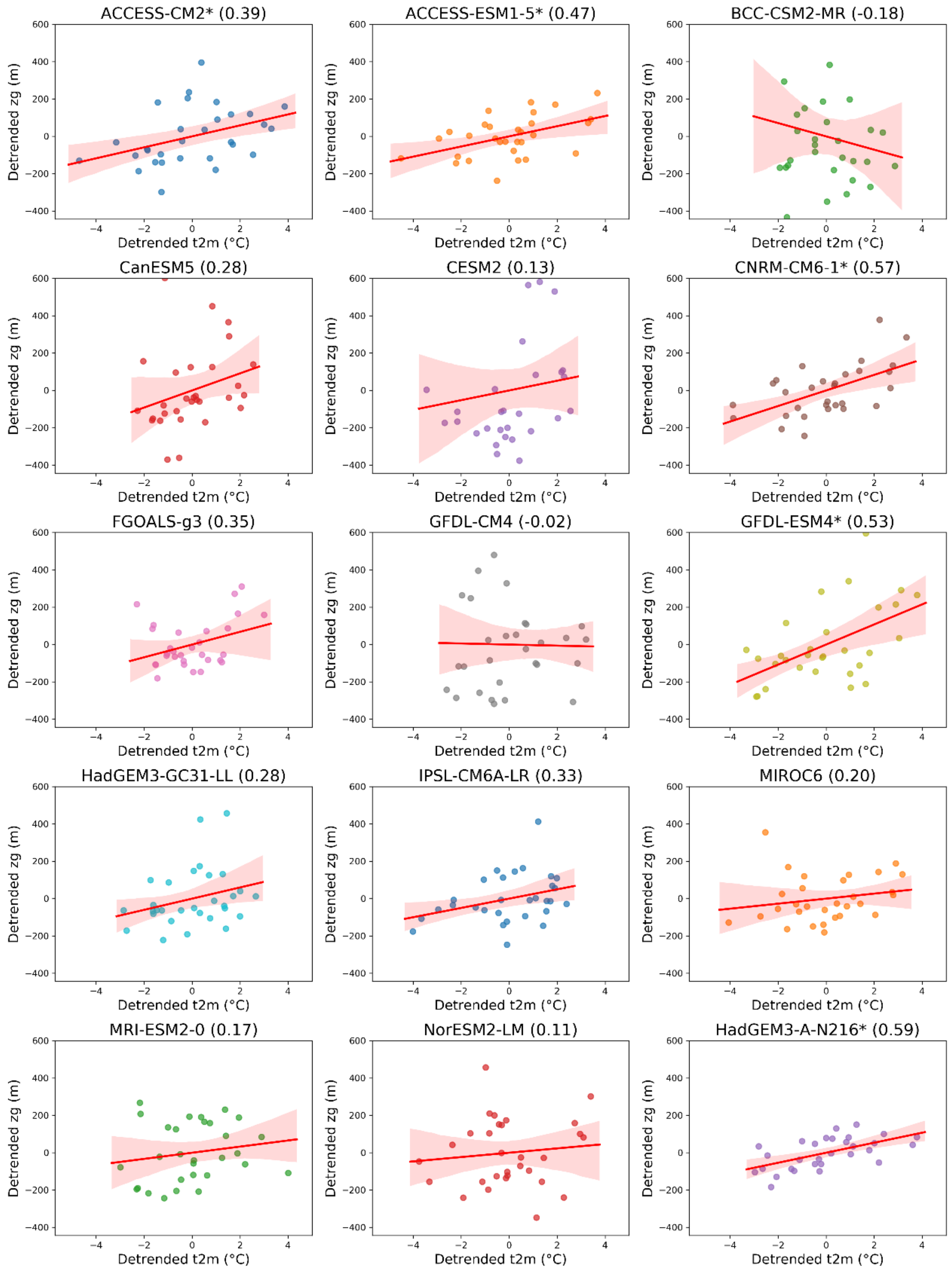

Supplementary Fig. 15 | Scatterplot between detrended T2m and 10 hPa geopotential height anomalies averaged over the study period and study region among 14 CMIP6 models and HadGEM3-A-N216 in the historical period. The values in parentheses indicate the respective correlation coefficients. The selected models are highlighted with asterisks (*).

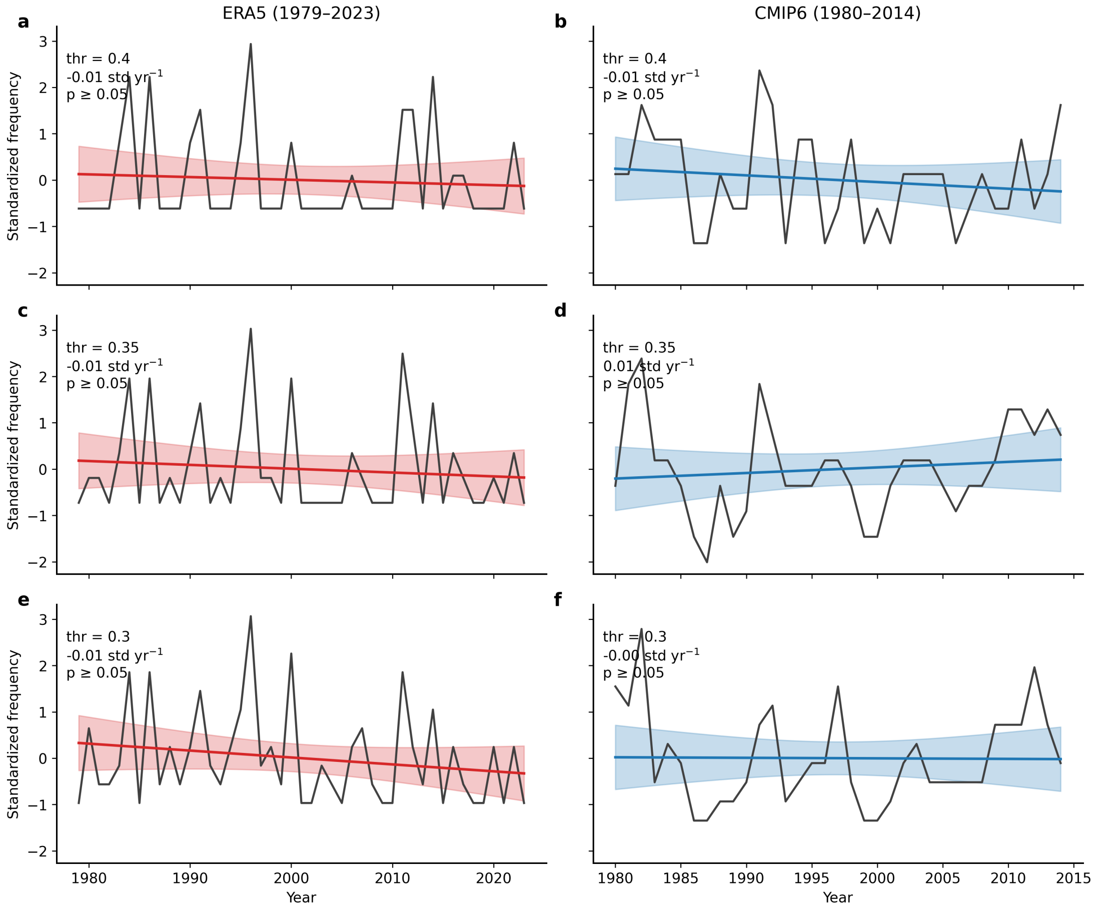

Supplementary Fig. 16 | Temporal evolution of 2024-like circulation analogue frequency. Standardized annual frequency of circulation patterns analogous to the peak day of the July–August 2024 Dronning Maud Land winter heatwave in (a, c, e) ERA5 (1979–2023) and (b, d, f) CMIP6 simulations (1980–2014), shown for three pattern correlation thresholds (0.4, 0.35, and 0.3). Solid lines denote linear regression trends, with shaded bands indicating the 95% confidence intervals. Analogues are identified based on daily 500 hPa and 10 hPa eddy geopotential height anomalies during July–August simultaneously exceeding the prescribed similarity threshold over the Antarctic region (90°S–60°S, 0°–360°E).

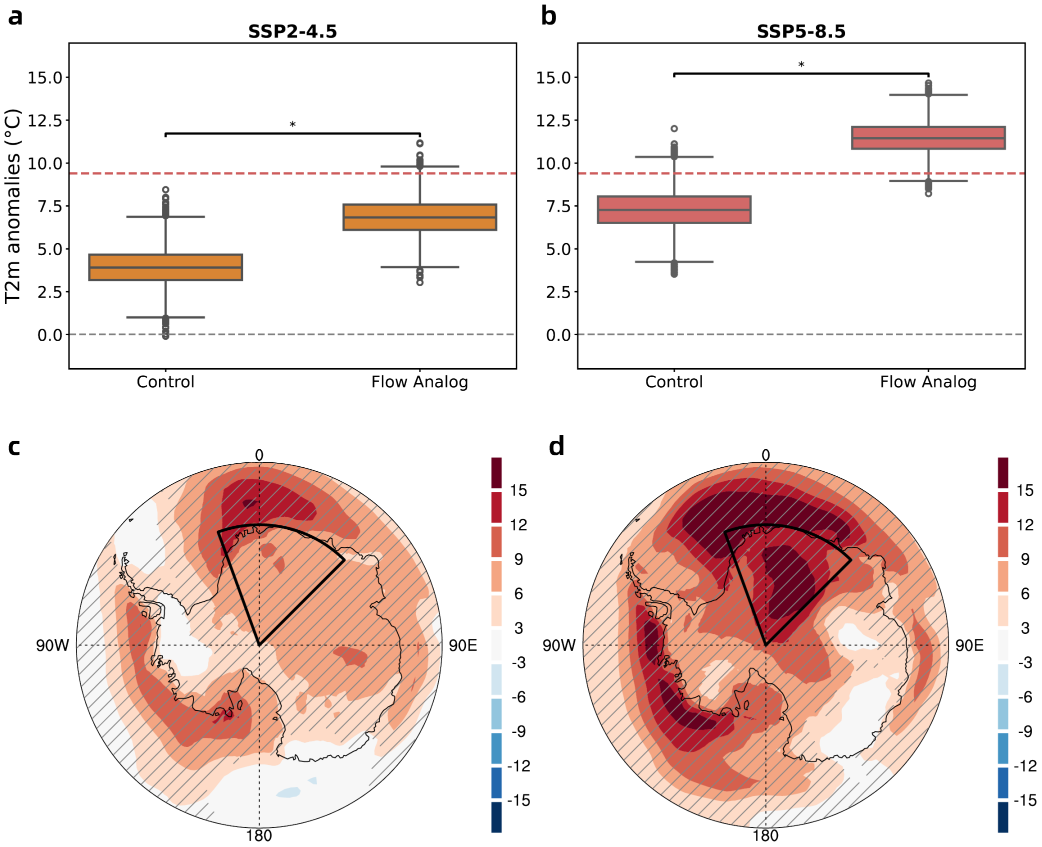

Supplementary Fig. 17 | (a, b) Distribution of T2m anomalies (unit: °C) on circulation analogue days compared to randomly selected control days for *SSP2-4.5* (orange) and *SSP5-8.5* (red) projections during 2080–2100. Each boxplot shows the interquartile range (25th–75th percentiles), with the central line denoting the median. The horizontal red dashed line represents the observed T2m anomaly of 2024 event derived from ERA5. Statistical significance of the differences between circulation analogue and control distributions is assessed using two-sided Mann–Whitney U tests, with asterisks (*) indicating p-values < 0.05. (c) Spatial distribution of T2m anomalies (unit: °C) on flow analogue days for *SSP2-4.5* and *SSP5-8.5* projections during 2080–2100, respectively. Hatchings indicate grid points where more than 90% of best-matched analogs show anomalies of the same sign.
